# Combined inhibition of EZH2 and ATM is synthetic lethal in BRCA1-deficient breast cancer

**DOI:** 10.1186/s13058-022-01534-y

**Published:** 2022-06-17

**Authors:** Leonie Ratz, Chiara Brambillasca, Leandra Bartke, Maxim A. Huetzen, Jonas Goergens, Orsolya Leidecker, Ron D. Jachimowicz, Marieke van de Ven, Natalie Proost, Bjørn Siteur, Renske de Korte-Grimmerink, Peter Bouwman, Emilia M. Pulver, Roebi de Bruijn, Jörg Isensee, Tim Hucho, Gaurav Pandey, Maarten van Lohuizen, Peter Mallmann, Hans Christian Reinhardt, Jos Jonkers, Julian Puppe

**Affiliations:** 1 Department of Obstetrics and Gynecology, University Hospital of Cologne, Kerpener Str. 34, 50931 Cologne, Germany; 2 Division of Molecular Pathology, Netherlands Cancer Institute, Amsterdam, The Netherlands; 3 Oncode Institute, Amsterdam, The Netherlands; 4 Max Planck Research Group Mechanisms of DNA Repair, Max Planck Institute for Biology of Ageing, Cologne, Germany; 5 Department I of Internal Medicine, Center for Integrated Oncology Aachen Bonn Cologne and Duesseldorf, Faculty of Medicine and University Hospital Cologne, University of Cologne, Cologne, Germany; 6 Cologne Excellence Cluster on Cellular Stress Responses in Ageing-Associated Diseases, University of Cologne, Cologne, Germany; 7 Center for Molecular Medicine Cologne, University of Cologne, Cologne, Germany; 8 Mouse Clinic for Cancer and Ageing, Netherlands Cancer Institute, Amsterdam, The Netherlands; 9 Division of Drug Discovery and Safety, Leiden Academic Centre for Drug Research, Leiden University, Leiden, The Netherlands; 10 Division of Molecular Carcinogenesis, Netherlands Cancer Institute, Amsterdam, The Netherlands; 11 Translational Pain Research, Department of Anaesthesiology and Intensive Care Medicine, University Hospital Cologne, Faculty of Medicine, University Cologne, Cologne, Germany; 12 Division of Molecular Genetics, Cancer Genomics Centre Netherlands, Netherlands Cancer Institute, Amsterdam, The Netherlands; 13 Department of Hematology and Stem Cell Transplantation, University Hospital Essen, University Duisburg-Essen, German Cancer Consortium (DKTK Partner Site Essen), Essen, Germany

**Keywords:** *BRCA1* mutation, Synthetic lethality, EZH2, Breast cancer

## Abstract

**Background:**

The majority of *BRCA1*-mutant breast cancers are characterized by a triple-negative phenotype and a basal-like molecular subtype, associated with aggressive clinical behavior. Current treatment options are limited, highlighting the need for the development of novel targeted therapies for this tumor subtype.

**Methods:**

Our group previously showed that EZH2 is functionally relevant in BRCA1-deficient breast tumors and blocking EZH2 enzymatic activity could be a potent treatment strategy. To validate the role of EZH2 as a therapeutic target and to identify new synergistic drug combinations, we performed a high-throughput drug combination screen in various cell lines derived from BRCA1-deficient and -proficient mouse mammary tumors.

**Results:**

We identified the combined inhibition of EZH2 and the proximal DNA damage response kinase ATM as a novel synthetic lethality-based therapy for the treatment of BRCA1-deficient breast tumors. We show that the combined treatment with the EZH2 inhibitor GSK126 and the ATM inhibitor AZD1390 led to reduced colony formation, increased genotoxic stress, and apoptosis-mediated cell death in BRCA1-deficient mammary tumor cells in vitro. These findings were corroborated by in vivo experiments showing that simultaneous inhibition of EZH2 and ATM significantly increased anti-tumor activity in mice bearing BRCA1-deficient mammary tumors.

**Conclusion:**

Taken together, we identified a synthetic lethal interaction between EZH2 and ATM and propose this synergistic interaction as a novel molecular combination for the treatment of *BRCA1*-mutant breast cancer.

**Supplementary Information:**

The online version contains supplementary material available at 10.1186/s13058-022-01534-y.

## Introduction

Triple-negative breast cancer (TNBC) is the most aggressive phenotype of breast cancer with limited treatment options and unfavorable prognosis. TNBCs encompass heterogeneous molecular subtypes, and 20–30% are associated with disabling germline *BRCA1/2* mutations (gBRCA) and a basal-like molecular subtype [1]. Moreover, 25% of sporadic TNBCs are characterized by (epi)genetic defects in homologous recombination (HR), that induce pathological and molecular changes resembling *BRCA1/2* mutation-associated cancers (BRCAness) [2–4]. TNBCs frequently show an inadequate response to (neo)adjuvant chemotherapy with poor survival benefit. Dose-dense neoadjuvant chemotherapy is highly recommended and the addition of carboplatin to the standard anthracycline- and taxane-based regimen of patients with TNBC increases the rate of pathological complete remission (pCR), a crucial surrogate of overall survival [5]. Highest pCR rates are achieved in patients with germline *BRCA1*-mutant breast cancer due to increased genomic instability, however, no overall survival benefit was observed [6–9]. Thus, a major effort in clinical breast cancer research is to define a therapeutic algorithm with novel treatments for patients with TNBC.

At the cellular level, the *BRCA1* gene product is important for DNA double-strand break (DSB) repair through the HR pathway. BRCA1-deficient cells display inappropriate DSB repair due to HR-deficiency. Consequently, those cells depend on the PARP1-mediated base excision repair pathway and non-homologous end joining (NHEJ) to resolve DNA damage, however, the error-prone character of NHEJ promotes chromosomal rearrangements and genomic instability. Checkpoint gene inactivation (by p53 inactivation, for example) disables cells to induce cell cycle arrest and further accelerates breast cancer development [10, 11]. Identifying the molecular pathogenesis of *BRCA1*-mutant TNBCs has directed the implementation of PARP inhibitors in locally advanced and metastatic breast cancer [12]. However, durable clinical response to PARP inhibition is counteracted by PARP inhibitor resistance [13]. The identification of novel molecular targets for a tailored treatment approach is thus of high importance to increase therapeutic options for patients with *BRCA1*-mutant breast cancer.

Recent work from our group and others has highlighted histone methyltransferase EZH2 (Enhancer of zeste homolog 2) as a promising target in BRCA1-deficient breast cancer [14–16]. EZH2 is the catalytic subunit of the Polycomb repressive complex 2 (PRC2) responsible for histone H3 lysine 27 trimethylation (H3K27me3) and transcriptional silencing of target genes. EZH2 is involved in various biological functions, including cell cycle control, proliferation, and differentiation in various cancer types [17, 18]. BRCA1 has been reported to be a negative regulator of EZH2 [15]. Loss of BRCA1 function was shown to increase EZH2 activity by promoting genome-wide EZH2 recruitment to chromatin. This caused an increase in H3K27me3 levels at known PRC2 target loci that have been associated with reduced cellular differentiation and an aggressive breast cancer phenotype [15]. EZH2 is overexpressed in tumors with loss of BRCA1 function or a *BRCA1*-like DNA copy number profile [14, 19, 20]. Patients with *BRCA1*-mutant breast cancer and high EZH2 expression showed an improved therapeutic response and less frequent disease recurrence after treatment with intensified platinum-based chemotherapy, compared to standard chemotherapy [14]. Combined treatment with the EZH2 inhibitor GSK126 and cisplatin led to increased cytotoxicity and reduced colony formation in BRCA1-deficient mouse mammary tumor cells [14]. Effective in vivo anti-tumor activity of combined GSK126 and cisplatin treatment also enhanced overall survival of mice bearing BRCA1-deficient mouse mammary tumors, suggesting that EZH2 inhibition enhances the sensitivity to platinum drugs in EZH2-overexpressing breast tumors [14].

Epigenetic synthetic lethal approaches have gained interest in cancer therapies, due to various aberrant epigenetic regulatory mechanisms leading to actionable dependencies [21]. Several synthetic lethal partners of EZH2 or mutations conferring EZH2 dependence have been identified in hematological malignancies and solid cancers, such as breast, ovarian, and non-small cell lung cancer [22–26]; reviewed by [21]. Since *BRCA1*-mutant breast cancers harbor a defect in their DNA repair machinery leading to a dependency on alternative repair pathways, synthetic lethal therapies show high potential in this genetic context as highlighted by the clinical use of PARP inhibitors to treat *BRCA1/2*-mutant breast and ovarian cancer [12, 27, 28]. We therefore aimed to identify synthetic lethal partners of EZH2 in BRCA1-deficient breast cancer.

## Results

### High-throughput drug screening identifies synergistic cytotoxicity of EZH2 and ATM inhibition in a BRCA1-deficient context

**Fig. 1:**
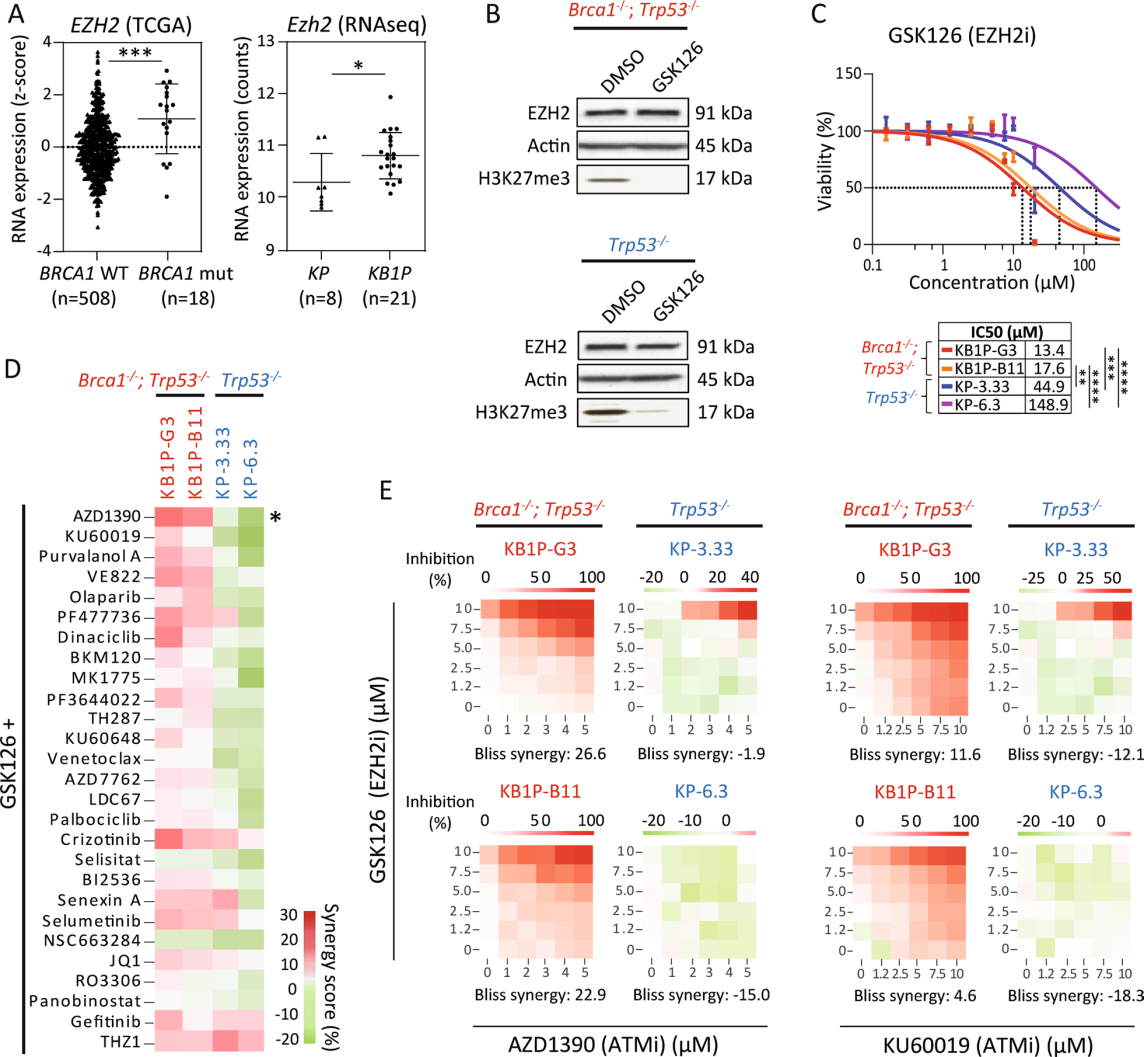
Synergistic cytotoxicity screen in combination with GSK126-induced EZH2 inhibition. **A** Increased *EZH2* mRNA expression in BRCA1-deficient breast cancer. Left panel: *EZH2* expression using the TCGA breast invasive carcinoma (BRCA) whole-exome sequencing data set (*n* = 826). Data are shown as *z*-score transformed in *BRCA1*-wild type (*n* = 508) versus *BRCA1*-mutated (*n* = 18) breast tumors. Right panel: *Ezh2* expression using RNAseq data from BRCA1-proficient KP (*n* = 8) and BRCA1-deficient KB1P mouse mammary tumors (*n* = 21). Groups were compared using an unpaired Mann–Whitney U test. **B** Target inhibition of H3K27me3 after 72 h of GSK126 treatment (5 µM) shown by a representative immunoblot in BRCA1-deficient KB1P-G3 and BRCA1-proficient KP-3.33 cells, β-actin served as loading control. **C** Single agent dose–response curves and determination of IC_50_ value of GSK126 in four mouse mammary tumor cell lines. Statistical significance was tested by one-way ANOVA with Tukey multiple comparison test. **D** Heatmap showing synergy scores calculated by the Bliss independence model of 27 compounds in combination with GSK126 across four murine mammary tumor cell lines after 72 h treatment. Combinations are arranged by the difference in synergy score between BRCA1-deficient and BRCA1-proficient cell lines from high to low. Cell lines are arranged by the *Brca1* mutation status. Concentrations and IC_50_ values of single compounds can be found in Additional file 8: Table S1. Asterix indicates the prioritized compound AZD1390. **E** Heatmaps visualized by the web-application tool SynergyFinder showing synergy scores of treatment combinations GSK126/AZD1390 and GSK126/KU60019, respectively. Displayed colors reflect the growth inhibition in percent with red indicating stronger inhibition and green indicating lower inhibition

Previously, we reported that EZH2 is highly expressed in tumors associated with a BRCAness profile. Here, the association of *EZH2* mRNA expression with *BRCA1-*mutant breast cancer was further evaluated using the TCGA BRCA whole exome sequencing data set (*n* = 526) confirming the increased expression of *EZH2* in *BRCA1*-mutant (*n* = 18), compared to *BRCA1*-wild type breast cancer (*n* = 508) (*p* = 0.0003; Fig. 1A, left panel). Significantly increased *Ezh2* expression in female BRCA1-deficient K14*cre*; *Brca1*^F/F^; *Trp53*^F/F^ (KB1P) mouse mammary tumors compared to BRCA1-proficient K14*cre*; *Trp53*^F/F^ (KP) mammary tumors was further corroborated by RNA sequencing of tumor tissue (*p* = 0.0156; Fig. 1A, right panel).

To study the role of EZH2 in *BRCA1*-mutant breast cancer, we made use of previously described murine tumor cell lines derived from KB1P (clones KB1P-G3 and KB1P-B11) or KP (clones KP-3.33 and KP-6.3) mammary tumors [29]. First, inhibition of the EZH2 methyltransferase activity by GSK126 was assessed, showing a reduced H3K27me3 level by immunoblotting (Fig. 1B). We then determined the IC_50_ value of GSK126 for all cell clones, showing that 3- to 11-fold lower concentrations of GSK126 were required for a 50% reduction in viability in BRCA1-deficient compared to BRCA1-proficient cells, indicating that BRCA1-deficient mammary tumor cells are more sensitive to EZH2 inhibition (Fig. 1C; KB1P-G3: IC_50_ = 13.4 µM; KB1P-B11: IC_50_ = 17.6 µM; KP-3.33: IC_50_ = 44.9 µM; KP-6.3: IC_50_ = 148.9 µM).

In order to identify synthetic lethal partners of EZH2 that are specifically effective in BRCA1-deficient breast cancer, we used BRCA1-deficient and -proficient mammary tumor cell lines to perform a high-throughput drug screen in combination with GSK126. We analyzed a panel of 27 distinct compounds targeting main oncogenic signaling pathways, such as DNA repair and cell cycle checkpoint signaling, and included small-molecule inhibitors that are in preclinical testing for cancer treatments. Single agent profiles for IC_50_ determination were assessed in serial dilutions for all compounds in BRCA1-deficient (KB1P-G3 and KB1P-B11) and BRCA1-proficient (KP-3.33 and KP-6.3) cells (Additional file 1: Fig. S1 and Additional file 8: Table S1). Each compound was then profiled for synergistic interaction with GSK126, using 6 × 6 concentration combinations (vehicle control, 5 single concentrations per compound, 25 combinations). For analysis of potential synergy, the Bliss independence model was used with a score of  >15 indicative of a synergistic compound interaction (Additional file 8: Table S1). Synergy scores were further analyzed based on the difference between BRCA1-deficient and BRCA1-proficient cell lines. Synergistic compound screening highlighted the two Ataxia telangiectasia mutated (ATM) inhibitors AZD1390 [30] and KU60019 [31] as the best hits with the highest difference in synergy scores between BRCA1-deficient and -proficient cells (Fig. 1D). Synergistic effects were visualized using the web-application tool SynergyFinder [32]. Heatmaps for GSK126/AZD1390 and GSK126/KU60019 combination treatments are depicted in Fig. 1E. Visualized synergy heatmaps and synergy scores for the remaining compounds in combination with GSK126 are shown in Additional file 2: Fig. S2 and Additional file 8: Table S1, respectively. Since the highest synergy score was observed for AZD1390 (Bliss synergy score: 26.6 in KB1P-G3, 22.9 in KB1P-B11), this ATM-kinase specific inhibitor was prioritized for further analysis in combination with GSK126.

ATM together with ATR (ataxia telangiectasia and Rad3 related) are DNA damage sensor kinases that, upon induction of DNA damage, phosphorylate checkpoint kinase 1 and 2 (CHK1/2), respectively [33]. This results in G1-S and G2-M cell cycle checkpoint activation and initiation of DNA repair. ATM is a central molecule in the signaling cascade of DSBs. In the absence of an intact DNA repair mechanism, collateral ATM-deficiency leads to increased genomic instability [34].

**Fig. 2:**
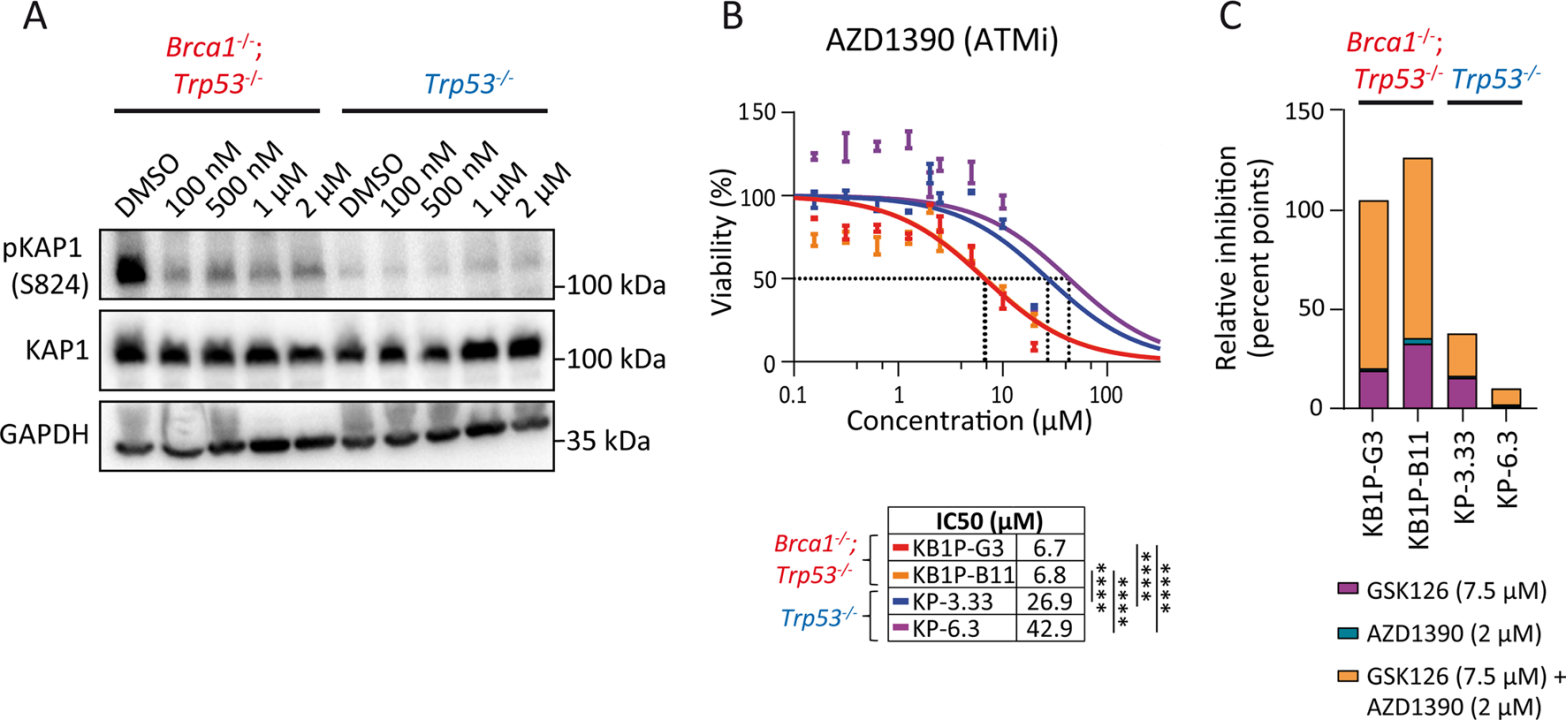
Validation of synergistic effect of EZH2/ATM inhibition. **A** Target inhibition of phosphorylated Kap1 (S824) after 72 h by increasing concentrations of AZD1390 (100 nM, 500 nM, 1 µM, 2 µM) treatment shown by a representative immunoblot in KB1P-G3 and KP-3.33 cells, GAPDH served as loading control. **B** Single agent dose–response curves and determination of IC_50_ value of AZD1390 in four mouse mammary tumor cell lines after 72 h treatment. **C** EZH2 inhibition specifically sensitizes BRCA1-deficient cells to ATM inhibition. The stacked bar graphs show the relative contribution to inhibition of cell viability by combined treatment with 7.5 µM GSK126/2 µM AZD1390 for 72 h. Statistical significance was tested by one-way ANOVA with Tukey multiple comparison test

The ATM inhibitor AZD1390 was able to inhibit downstream ATM signaling as shown by reduced Kap1 (S824) phosphorylation at various doses (0.1–2 µM) in BRCA1-deficient and BRCA1-proficient cells (Fig. 2A). However, AZD1390 showed 4- to 6-fold lower IC_50_ values for BRCA1-deficient cells than BRCA1-proficient cells, indicating that BRCA1-deficient cells are more sensitive to ATM inhibition (Fig. 2B; KB1P-G3: IC_50_ = 6.70 µM; KB1P-B11: IC_50_ = 6.8 µM; KP-3.33: IC_50_ = 26.9 µM; KP-6.3: IC_50_ = 42.90 µM).

### In vitro optimization of the synergistic effect from combined EZH2/ATM inhibition

In addition to genetic context, the choice of compound concentration is crucial to achieve maximal drug synergy and to avoid over- or under-treatment resulting in unspecific toxicity or response failure [35]. Therefore, a concentration optimization experiment was performed to determine the compound concentration with the highest synergy between GSK126 and AZD1390. BRCA1-deficient and -proficient mouse mammary tumor cells were treated with increasing concentrations of one compound in the absence or presence of a fixed concentration of the other compound and vice versa (Additional file 3: Fig. S3). Single agent GSK126 treatment at intermediate concentrations induced moderate cytotoxicity in BRCA1-deficient cells, but strong cytotoxicity with the highest concentration of 10 µM (Additional file 3: Fig. S3A, panels in second row). The cytotoxic effect could be potentiated by adding a fixed concentration of AZD1390, while this concentration had no cytotoxic effect as a single treatment (Additional file 3: Fig. S3B). Treatment with increasing concentrations of single agent AZD1390 slightly decreased BRCA1-deficient cell viability, while no cytotoxic effect was observed in BRCA1-proficient cells (Additional file 3: Fig. S3B). When introducing GSK126 at a fixed concentration, BRCA1-deficient cells were sensitized to the cytotoxic effect of AZD1390, while BRCA1-proficient cells remained unaffected by this combination. As was evident from the IC_50_ determination, high concentrations of single GSK126 or AZD1390 treatment also induced toxicity in BRCA1-proficient cells (Figs. 1C, 2B), however, this cytotoxicity was not attributed to a synergistic effect but rather to single agent-induced toxicity. This experiment revealed maximal synergistic interaction between 7.5 µM GSK126 and 3 µM AZD1390 in BRCA1-deficient cells (Additional file 3: Fig. S3A, panels in third row and Fig. S3B, panels in fourth row). Importantly, this combination treatment also induced slight cytotoxicity in BRCA1-proficient cells. We therefore used 7.5 µM GSK126 and 2 µM AZD1390 in subsequent validation experiments, which is below the half-maximal inhibitory concentration of both compounds. First, the inhibitory effect on cell viability was validated in an independent cell viability measurement using CellTiter-Glo (Fig. 2C). GSK126 and AZD1390 single agent treatment induced 17% and 7% reduction of viability in BRCA1-deficient cells, respectively. Remarkably, the combination of both drugs displayed 93% cytotoxicity in BRCA1-deficient cells (Fig. 2C), while no significant toxicity was observed in BRCA1-proficient cells, confirming that the reduction in cell viability was triggered by context-specific synergistic activity of GSK126 and AZD1390 in BRCA1-deficient cells.

**Fig. 3:**
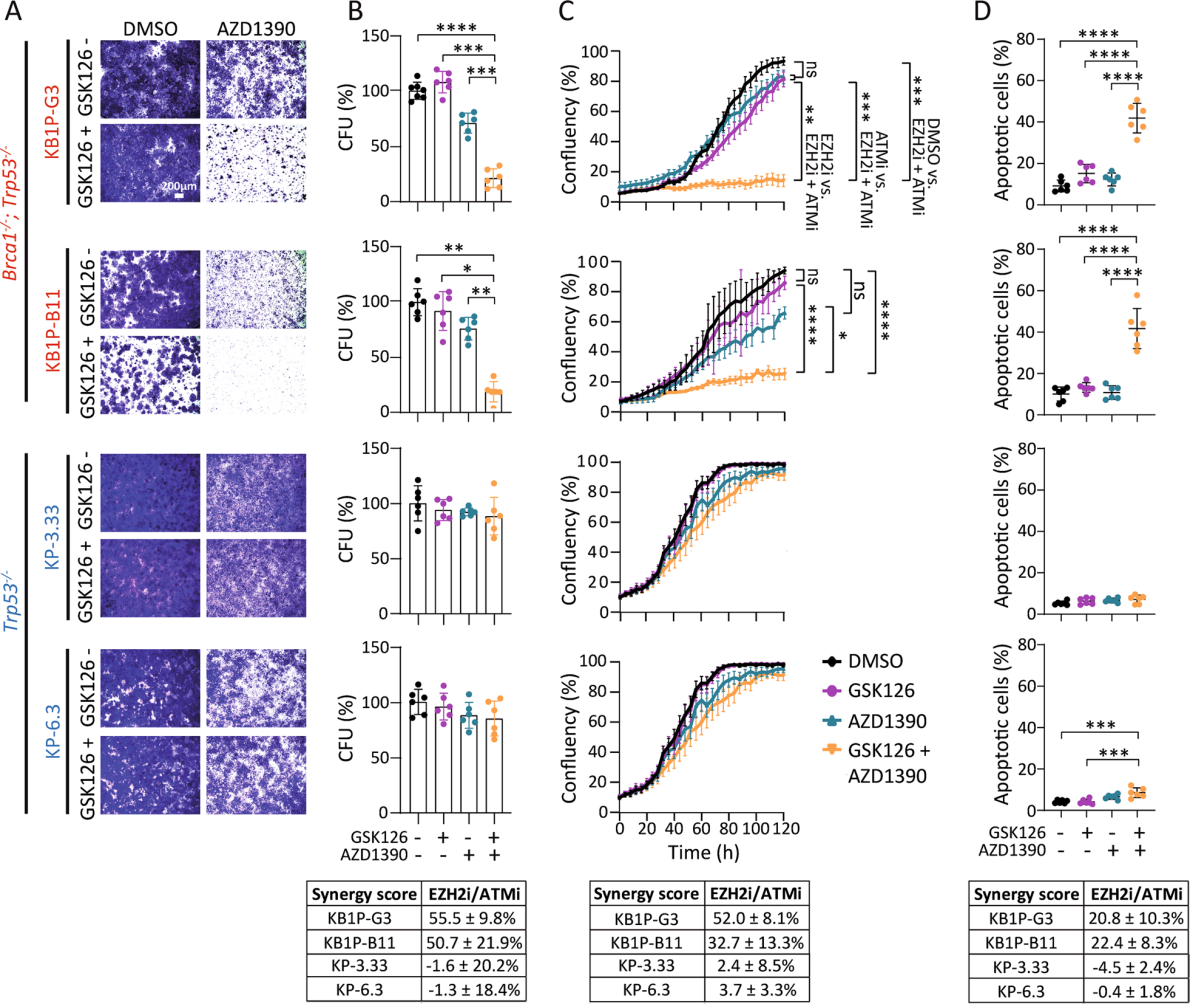
Functional characterization of combined EZH2/ATM inhibition. **A**, **B** Colony formation assay in BRCA1-deficient (KB1P-G3 and KB1P-B11) and BRCA1-proficient (KP-3.33 and KP-6.3) mouse mammary tumor cells after 7 days of incubation. **A** Representative microscopic images of colony formation at 0.65× magnification. Scale bar 200 µm. **B** Quantification of crystal violet positive stained cells is presented as mean ± SD of six independent experiments. Calculation of drug synergy using the Bliss independence score revealed a mean synergy score of 55.5 ± 9.8% in KB1P-G3, 50.7 ± 21.9% in KB1P-B11, −1.6 ± 20.2% in KP-3.33, and −1.3 ± 18.4% in KP-6.3 cells. **C** Growth inhibition curves using IncuCyte live cell imaging measured by cell densities on culture plates from at least three independent experiments with each three technical replicates. Bliss synergy score: KB1P-G3: 52.0 ± 8.1%; KB1P-B11: 32.7 ± 13.3%; KP-3.33: 2.4 ± 8.5%; KP-6.3: 3.7 ± 3.3%. **D** Flow cytometric analysis of apoptotic cells measured by Annexin V and propidium iodide double staining of cells treated with GSK126 and AZD1390 for 48 h. Bliss synergy score: KB1P-G3: 20.8 ± 10.3%; KB1P-B11: 22.4 ± 8.3%; KP-3.33: −4.5 ± 2.4%; KP-6.3: −0.4 ± 1.8%. All experiments were performed using inhibitor concentrations of 7.5 µM GSK126 and 2 µM AZD1390. Significance was tested by one-way ANOVA with Tukey multiple comparison test. *CFU* colony forming unit

Combined EZH2/ATM inhibitor treatment was subsequently validated using different functional assays. Assessment of clonogenic survival revealed a substantial decrease in the number of colonies by combined GSK126/AZD1390 treatment in BRCA1-deficient mouse mammary tumor cells, compared to vehicle control, as well as to single GSK126 or AZD1390 treatment (Fig. 3A). Quantification of colony formation showed that combined GSK126/AZD1390 treatment caused a 79–81% decrease in colony-forming units in BRCA1-deficient cells compared to vehicle control (KB1P-G3: *p* < 0.0001, KB1P-B11: *p* = 0.002), a 73–86% decrease compared to single GSK126 treatment (KB1P-G3: *p* = 0.0001, KB1P-B11: *p* = 0.01), and a 50–57% decrease compared to single AZD1390 treatment (KB1P-G3: *p* = 0.0001, KB1P-B11: *p* = 0.001), but not in BRCA1-proficient cells, indicating a robust synergistic effect in BRCA1-deficient cells (Fig. 3B). Continuous live-cell imaging using IncuCyte analysis revealed that growth inhibition in BRCA1-deficient cells through combined GSK126/AZD1390 treatment occurred between 30–40 h after induction of treatment, resulting in 68–79% growth reduction after 120 h in BRCA1-deficient cells, compared to vehicle control (KB1P-G3: *p* < 0.0001, KB1P-B11: *p* < 0.0001) (Fig. 3C), while growth was not affected in BRCA1-proficient cells. Single agent treatment showed a mild growth inhibitory effect in BRCA1-deficient cells, which was not statistically significant compared to vehicle control.

### Cytotoxicity by combined EZH2/ATM inhibition is mediated by apoptosis

To investigate whether combined EZH2/ATM inhibition induces apoptosis in BRCA1-deficient mouse mammary tumor cells, we measured the percentage of Annexin V/propidium iodide double-positive cells by flow-cytometry (Fig. 3D). Indeed, the percentage of apoptotic cells drastically increased by combined GSK126/AZD1390 inhibition compared to vehicle control or single agent treatment in BRCA1-deficient cells (KB1P-G3: 41.8% by GSK126/AZD1390 compared to 9.1% by vehicle (*p* < 0.0001), 15.1% by GSK126 (*p* < 0.0001) and 12.3% by AZD1390 (*p* < 0.0001); KB1P-B11: 41.7% by GSK126/AZD1390 compared to 10.1% by vehicle (*p* < 0.0001), 13.3% by GSK126 (*p* < 0.0001) and 10.8% by AZD1390 (*p* < 0.0001)). In contrast, the percentage of apoptotic cells in BRCA1-proficient cells did not change significantly upon combined EZH2/ATM inhibition compared to single agent treatment or vehicle control (KP-3.33: 7.2% by GSK126/AZD1390 compared to 5.3% by vehicle (*p* = 0.17), 6.3% by GSK126 (*p* = 0.78) and 6.7% by AZD1390 (*p* = 0.95); KP-6.3: 8.5% by GSK126/AZD1390 compared to 4.2% by vehicle (*p* = 0.0004), 4.2% by GSK126 (*p* = 0.0004, showing a statistically significant, however, biologically insignificant difference) and 6.6% by AZD1390 (*p* = 0.14) (Fig. 3D). Together, these results indicate that the synergistic cytotoxicity of combined EZH2/ATM inhibition in BRCA1-deficient mouse mammary tumor cells is mediated by apoptosis.

### Cytotoxic effect of combined EZH2/ATM inhibition is evident in human TNBC cells

**Fig. 4:**
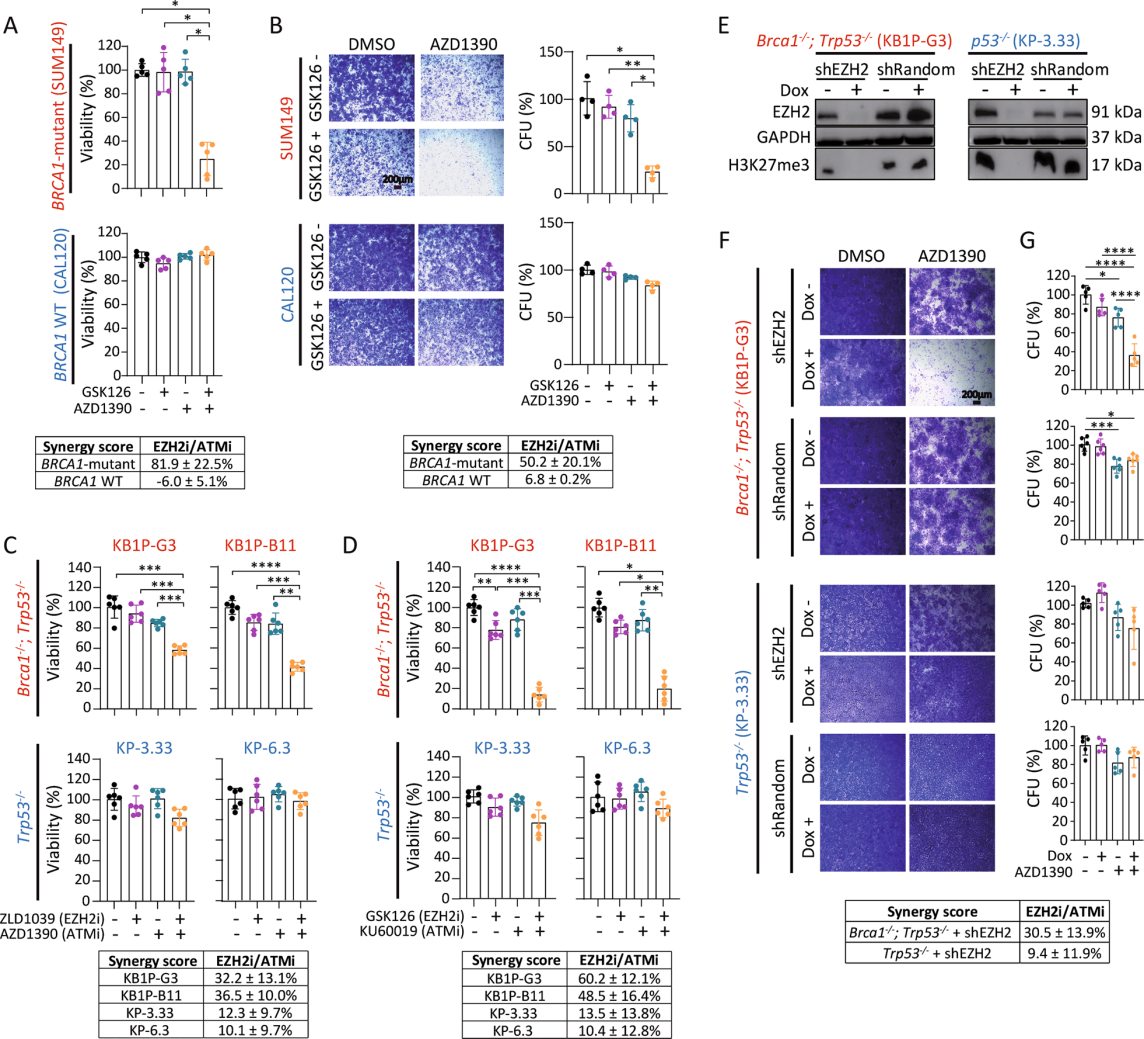
Validation of synergistic EZH2/ATM inhibition using human TNBC cells, chemically distinct inhibitors and genetic knockdown. **A** Cell viability assay showing increased cytotoxicity using combined GSK126 (7.5 µM) and AZD1390 (2.5 µM) for 72 h in *BRCA1*-mutant SUM149 (upper panel, Bliss synergy score: 81.9 ± 22.5%) compared to *BRCA1*-wild type CAL120 (lower panel, synergy score: −6.0 ± 5.1%) human breast cancer cells. Bars presented as mean ± SD of five independent experiments. **B** Colony formation assay in *BRCA1*-mutant (SUM149) and *BRCA1*-wild type (CAL120) human TNBC cells after 7 days of incubation with GSK126 (7.5 µM) and AZD1390 (2.5 µM). Left panels: Representative microscopic images of colony formation at 0.65× magnification. Scale bar 200 µm. Right panels: Quantification of crystal violet positive stained cells is presented as mean ± SD of four independent experiments. Calculation of drug synergy using the Bliss independence score revealed a mean synergy score of 50.2 ± 20.1% in SUM149 cells, 6.8 ± 0.2% in CAL120 cells. **C**, **D** The synergistic cytotoxic effect is also evident for chemically distinct inhibitors of EZH2 and ATM. The BRCA1-deficient (KB1P-G3 and KB1P-B11) and BRCA1-proficient (KP-3.33 and KP-6.3) cell lines were treated with **C** ZLD1039 (3 µM) as an alternative inhibitor against EZH2 alone or in combination with AZD1390 (5 µM), or **D** KU60019 (5 µM) as an alternative inhibitor against ATM alone or in combination with GSK126 (7.5 µM), respectively. After 72 h, cell viability was quantified by CellTiter-Glo assay. Data are presented as mean ± SD of at least four independent experiments. Bliss synergy score for ZLD1039/AZD1390: KB1P-G3: 32.2 ± 13.1%; KB1P-B11: 36.5 ± 10.0%; KP-3.33: 12.3 ± 9.7%; KP-6.3: 10.1 ± 9.7%. Bliss synergy score for GSK126/KU60019: KB1P-G3: 60.2 ± 12.1%; KB1P-B11: 48.5 ± 16.4%; KP-3.33: 13.5 ± 13.8%; KP-6.3: 10.4 ± 12.8%. **E**–**G** Genetic ablation of EZH2 mimics pharmacological H3K27me3 inhibition. **E** Immunoblotting showing reduced EZH2 expression and reduced H3K27me3 levels upon inducible *EZH2* knockdown after 7 days of Dox treatment (100 ng/µl) compared to PBS control treatment. **F** Representative microscopic images of colony formation assay after 7 days of incubation using Dox-induced *EZH2* knockdown and 2 µM AZD1390 in KB1P-G3 and KP-3.33-shEZH2 cells are shown at 0.65× magnification. Scale bar 200 µm. **G** Quantification of crystal violet positive stained colonies is presented as mean ± SD of five independent experiments. Bliss synergy score: KB1P-G3-shEZH2: 30.5 ± 13.9%; KP-3.33-shEZH22: 9.4 ± 11.9%. Significance was tested by one-way ANOVA with Tukey multiple comparison test. *CFU* colony forming unit

To investigate the cytotoxic effect of combined EZH2 and ATM inhibition in human *BRCA1*-mutant breast cancer, we treated the human TNBC cell lines SUM149 (*BRCA1*-mutant) and CAL120 (*BRCA1*-wild type) with GSK126 and AZD1390, and analyzed cell viability using CellTiter-Glo and clonogenic survival (Fig. 4A, B). SUM149 cells were specifically sensitive to combined GSK126/AZD1390 inhibition leading to a significantly reduced cell viability by 77–79%, compared to vehicle control (*p* = 0.02) or single agent treatment (GSK126: *p* = 0.02; AZD1390: *p* = 0.03), while no effect was observed in CAL120 cells (Fig. 4A), suggesting that the cytotoxic effect by EZH2/ATM inhibition might be specific to *BRCA1* mutation status in human breast cancer cells. Again, assessment of clonogenic survival revealed a significant decrease in the number of colonies by combined GSK126/AZD1390 treatment in *BRCA1*-mutant TNBC cells, compared to vehicle control, as well as to single GSK126 or AZD1390 treatment (Fig. 4B). Quantification of colony formation revealed that combined GSK126/AZD1390 treatment caused a 78% decrease in colony-forming units in *BRCA1*-mutant SUM149 cells compared to vehicle control (SUM149: *p* < 0.015), a 69% decrease compared to single GSK126 treatment (SUM149: *p* = 0.004), and a 57% decrease compared to single AZD1390 treatment (SUM149: *p* = 0.012), but not in *BRCA1*-wild type CAL120 cells, corroborating a robust synergistic effect also in human *BRCA1*-mutant TNBC cells (Fig. 4B).

### Cytotoxic effect of combined EZH2/ATM inhibition is stable after compound exchange

To confirm that the observed treatment effect is due to combined EZH2/ATM inhibition and not caused by any potential off-target effect, BRCA1-deficient and -proficient mouse mammary tumor cells were treated with the structurally distinct EZH2 and ATM inhibitors ZLD1039 [36] and KU60019 [31], respectively (Fig. 4C, D). Combined EZH2/ATM inhibition by GSK126/KU60019 or ZLD1039/AZD1390 treatment reduced the viability of BRCA1-deficient cells compared to vehicle control by 80–90% (KB1P-G3: *p* < 0.0001, KB1P-B11: *p* = 0.017), and by 42–59% (KB1P-G3: *p* = 0.0005, KB1P-B11: *p* < 0.0001), respectively, whereas growth of BRCA1-proficient cells was not significantly affected by either combination. These data indicate that the cytotoxic effects of the drug combinations are resultant of the combined EZH2/ATM inhibition and not due to unspecific compound effects.

### Genetic ablation of EZH2 mimics pharmacological H3K27me3 inhibition

To further establish the role of EZH2 inhibition in sensitizing BRCA1-deficient cells to ATM inhibition and to exclude off-target drug activity, we made use of a doxycycline (Dox)-inducible shRNA-mediated knockdown system [37] allowing stable and inducible *EZH2* knockdown in BRCA1-deficient (KB1P-G3-shEZH2) and BRCA1-proficient (KP-3.33-shEZH2) cells. Following seven days of Dox-induced *EZH2* knockdown, a strong decrease in EZH2 protein expression and global H3K27 trimethylation was observed in shEZH2-expressing cells but not in cells expressing a Dox-inducible random shRNA (shRandom) sequence (Fig. 4E). Next, clonogenic growth was assessed upon Dox-induced *EZH2* knockdown alone or in combination with the ATM inhibitor AZD1390 (Fig. 4F, G). In BRCA1-deficient cells, *EZH2* knockdown combined with AZD1390 induced 64% reduction in clonogenic growth compared to vehicle control (KB1P-G3-shEZH2: *p* < 0.0001), while shRandom-expression in combination with AZD1390 had no effect (Fig. 4F, G, upper two panels). In BRCA1-proficient cells, a mild effect was observed upon *EZH2* knockdown in combination with AZD1390, however, this effect was not statistically significant (KP-3.33-shEZH2: *p* = 0.12; Fig. 4F, G, lower two panels).

### Combined EZH2/ATM inhibition induces genotoxic stress

**Fig. 5:**
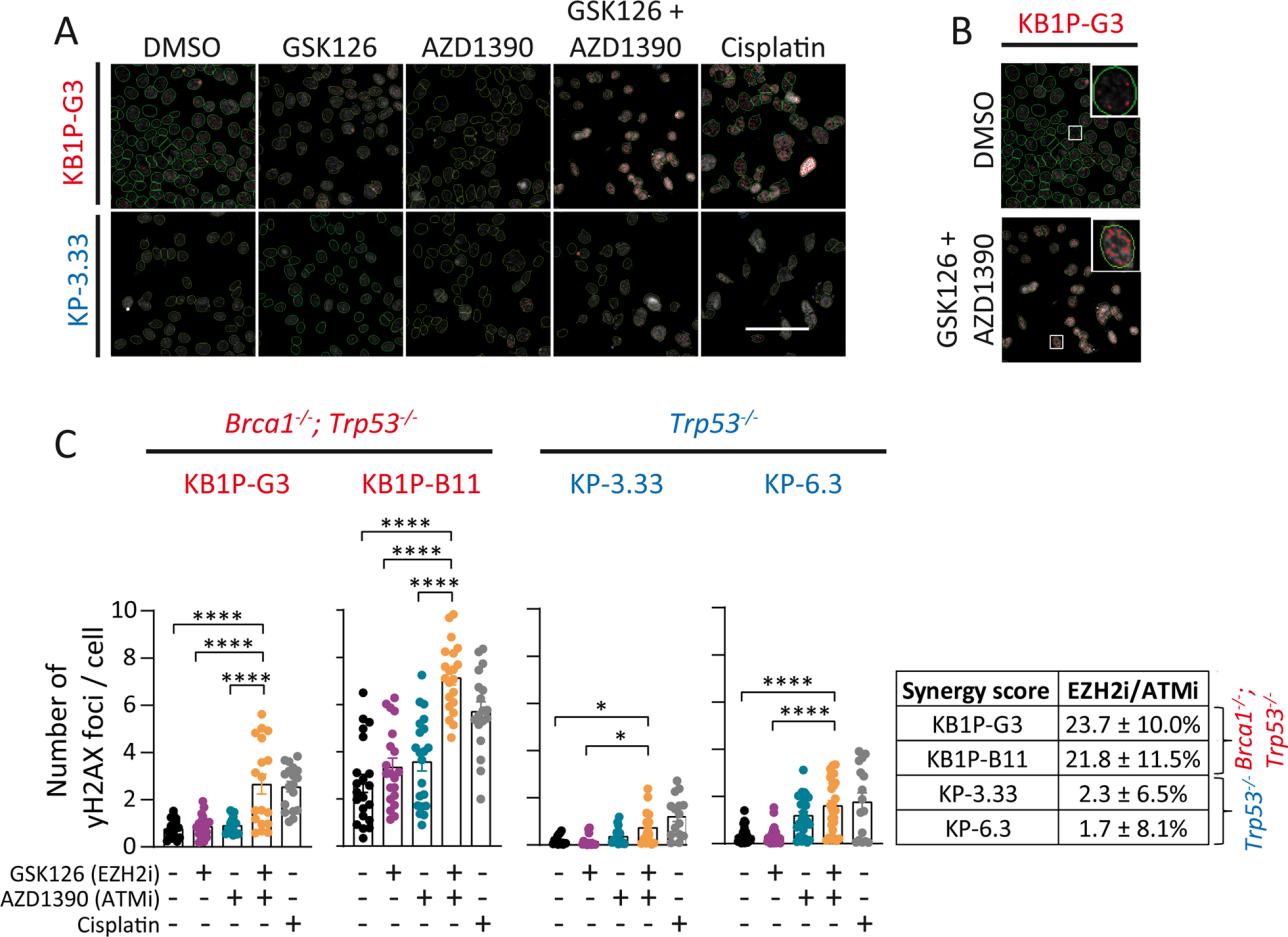
Combined inhibition of EZH2/ATM induces genotoxic stress. **A** Representative microscopic images of immunofluorescence staining of γH2AX in the nucleus of cells exposed to 7.5 µM GSK126 and 2 µM AZD1390 for 48 h. Treatment with 1 µM cisplatin was used as positive control. Green or blue encircled cells indicate inclusion or exclusion of objects. Red indicates detected yH2AX foci. Scale bar 100 µm. **B** Magnified inset of a representative KB1P-G3 cell with (top) high number of yH2AX foci per cell after combined EZH2/ATM inhibition and (bottom) low number of yH2AX foci per cell after DMSO treatment. **C** Quantification of immunofluorescence staining. Values are mean ± SEM of at least four independent experiments with each three technical replicates. Bliss synergy score: KB1P-G3: 23.7 ± 10.0%; KB1P-B11: 21.8 ± 11.5%; KP-3.33: 2.3 ± 6.5%; KP-6.3: 1.7 ± 8.1%. See Additional file 4: Fig. S4 for bar graphs showing cells per field (upper panels) and numbers of analyzed cells per condition (lower panels). Significance was tested by one-way ANOVA with Tukey multiple comparison test

We hypothesized that the observed increase in apoptotic cell death in BRCA1-deficient cells by combined EZH2/ATM inhibition was driven by higher levels of DNA damage. Immunoblotting showed inhibition of phospho-ATM and downstream phospho-Kap1 after AZD1390 single and combination treatment with GSK126, which is the expected effect of AZD1390-mediated ATM inhibition (Additional file 5: Fig. S5). However, immunoblotting is not sufficiently sensitive to detect slight changes in phosphorylated nuclear histone H2AX (yH2AX)yH2AX foci [38]. We used immunofluorescence staining as a more sensitive technique to detect yH2AX changes, together with the previously established automated high-throughput microscopy [39] to improve accuracy for counting of yH2AX foci formation and to assess the level of DNA damage and repair ability in BRCA1-deficient and BRCA1-proficient mouse mammary tumor cells treated with GSK126 and AZD1390 (Fig. 5A, B). The number of yH2AX foci per cell, which correspond with sites of DSBs, was highest in KB1P-G3 and KB1P-B11 cells treated with combined GSK126/AZD1390, showing a two to threefold increase, compared to vehicle control or single agent treatment (Fig. 5C). We also detected a slight, but statistically significant increase of yH2AX foci per cell upon combined GSK126/AZD1390 treatment in KP-3.33 and KP-6.3 cells, which, however, does not correlate with increased cytotoxicity (Fig. 3). Cisplatin (1 µM) served as a positive control and brought about a two to threefold increased number of yH2AX foci per cell as a result of DNA adduct formation. As expected, the number of detected cells per field was lowest in GSK126/AZD1390 and cisplatin-treated BRCA1-deficient cells (Additional file 4: Fig. S4). These results indicate that combined EZH2/ATM inhibition induces increased DNA damage in BRCA1-deficient mouse mammary tumor cells.

### Combination effect of EZH2/ATM inhibition in BRCA1-deficient mouse mammary tumor allografts

**Fig. 6:**
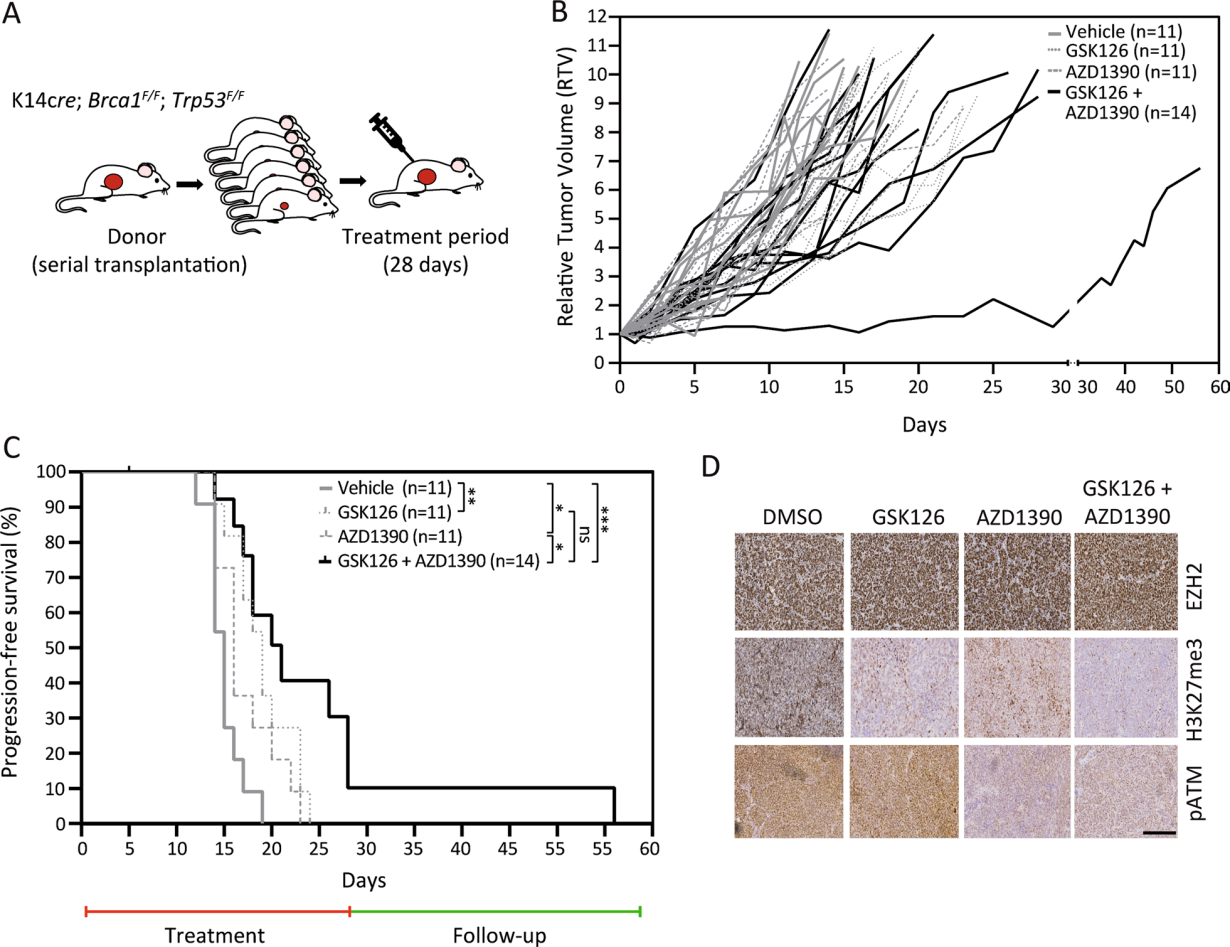
Combined EZH2/ATM inhibition shows an enhanced anti-tumor effect in KB1P mammary tumor allografts. **A** Mammary tumor tissue fragments from KB1P mice were transplanted into the fourth mammary fat pad of FVB females and treatments were initiated following tumor outgrowth to approximately 100 mm^3^ (100%). Upon tumor detection (day 0), mice were treated for 28 consecutive days. **B** Relative tumor volume curves. Tumor size was measured twice a week. For a better comparability, the tumor volume at day x was normalized to the initial tumor volume at treatment start (day 0) and defined as relative tumor volume (RTV). **C** Progression-free survival curves of KB1P mammary tumor-bearing mice treated with vehicle (grey), GSK126 (150 mg/kg i.p.) single agent (dotted line), AZD1390 (2 × 20 mg/kg, oral gavage) single agent (dashed line), or combined treatment with GSK126/AZD1390 (black). Progression-free survival was defined as the time to develop a 10-time increase in tumor volume. Survival curves were generated with the Kaplan–Meier approach and compared with the log-rank test as indicated. Censored animals (*n* = 3) are indicated by tick marks. **D** Representative images of immunofluorescence staining of EZH2, H3K27me3 and phosphorylated ATM in mammary tumor tissue after treatment as indicated. Scale bar 200 µm

To test the effect of combined EZH2/ATM inhibition in vivo, mice bearing tumor fragments derived from KB1P donor mice (Fig. 6A) were treated with GSK126 and AZD1390. Dose optimization was performed prior to survival analysis (Additional file 6: Fig. S6). For drug treatment (Fig. 6), mice were treated with vehicle, GSK126 (150 mg/kg, by daily intraperitoneal injection), AZD1390 (20 mg/kg, by oral gavage twice daily, 5 days on, 2 days off) or the combination of GSK126 and AZD1390 for 28 consecutive days, as evaluated previously [14, 30]. We observed a modest effect on tumor growth by single GSK126 or AZD1390 treatment. However, after combined GSK126/AZD1390 therapy tumor growth was reduced compared to the single agents (Fig. 6B). As a result of the treatment, progression-free survival was significantly increased by single agent treatment using GSK126 compared to vehicle control (*p* = 0.002), as wells as with AZD1390 treatment compared to vehicle control (*p* = 0.039). The longest progression-free survival was observed in the GSK126/AZD1390 combination therapy cohort, which was significantly increased compared to vehicle (*p* = 0.0001), and to single agent treatment with AZD1390 (*p* = 0.015) (Fig. 6C). Drug treatment was well tolerated in vivo and showed no toxicity to the animals (Additional file 7: Fig. S7). Immunohistochemistry analysis of GSK126 and AZD1390 treated tumors confirmed effective target inhibition by reduced H3K27me3 and by phosphorylated ATM levels, respectively (Fig. 6D). The results of double agent treatment in vivo suggest a combination effect by GSK126/AZD1390 treatment and support our in vitro findings of increased sensitivity in BRCA1-deficient mammary tumor cells.

## Discussion

The identification of targeted treatment strategies for BRCA1-deficient breast cancer is a current emphasis of preclinical research and clinical practice. Using a high-throughput drug screen, we identified synergy between inhibition of EZH2 and ATM with specific cytotoxicity in BRCA1-deficient tumor cells, thereby providing a molecularly rationalized approach that could guide clinical investigations of a combined therapy with EZH2 and ATM inhibitors in BRCA1-deficient breast cancer. The synergy of a combined EZH2/ATM inhibition in BRCA1-deficient tumor cells could be demonstrated by using several small molecule inhibitors of EZH2 (GSK126, ZLD1039) and ATM (AZD1390, KU60019) that are currently at various stages of preclinical development. Targeting ATM signaling is currently evaluated in HR-deficient breast cancer in several phase I and II trials [34, 40], corroborating the clinical significance of our approach. The synergistic cytotoxic effect of combined EZH2/ATM inhibition could further be confirmed by genetic downregulation of *EZH2* in combination with pharmacological ATM inhibition. Our in vivo study shows that the combined treatment with GSK126 and AZD1390 confers stronger anti-tumor activity than either inhibitor alone in BRCA1-deficient mammary tumors in mice, although the effect on progression-free survival was not strong.

GSK126, one of the first selective EZH2 inhibitors, was extensively tested in DLBCL lymphoma cell lines leading to significant growth inhibition and increased apoptotic rate with maximal potency after 2 days [41]. GSK126 administration in preclinical xenograft models showed tumor stasis with 50 mg/kg once daily, and even complete tumor eradication with 150 mg/kg [41]. Single GSK126 treatment of the TNBC cell line MDA-MB-231 induced only modest inhibition of cell survival (concentrations up to 8 µM), while the combination of GSK126 and gefitinib was synergistic on apoptosis-mediated cell death [42]. Phase I clinical trials with single GSK126 treatment in non-hodgkin-lymphoma and multiple myeloma had been discontinued due to insufficient therapeutic activity (ClinicalTrials.gov: NCT02082977) [43]. Presumably, inhibition of EZH2-associated H3K27me3 methylation rather enhances sensitivity to other agents by reversing gene silencing. Therefore, novel treatment regimens combining GSK126 with other compounds targeting key signaling pathways or administering GSK126 in cancers harboring synthetic lethal mutations are currently under preclinical investigation and could be evaluated in future clinical trials [21, 44, 45]. Recent studies have shown that EZH2 could have H3K27me3-independent effects on gene regulation and cellular functions (reviewed in [46]). Thus, targeting its noncanonical function in EZH2 overexpressing tumors could lead to the development of more effective therapeutics.

The ATM kinase inhibitor AZD1390 has been tested in preclinical models of glioblastoma multiforme (GBM) and lung cancer as sensitizer to radiotherapy [30]. Interestingly, AZD1390 preferentially radiosensitized p53-deficient GBM cells. P53-deficiency disabled GBM cells to induce cell cycle arrest upon radiation-induced DNA damage, which was further exacerbated by AZD1390-mediated ATM inhibition and subsequent failure of DNA damage repair leading to cell death [30].

**Fig. 7:**
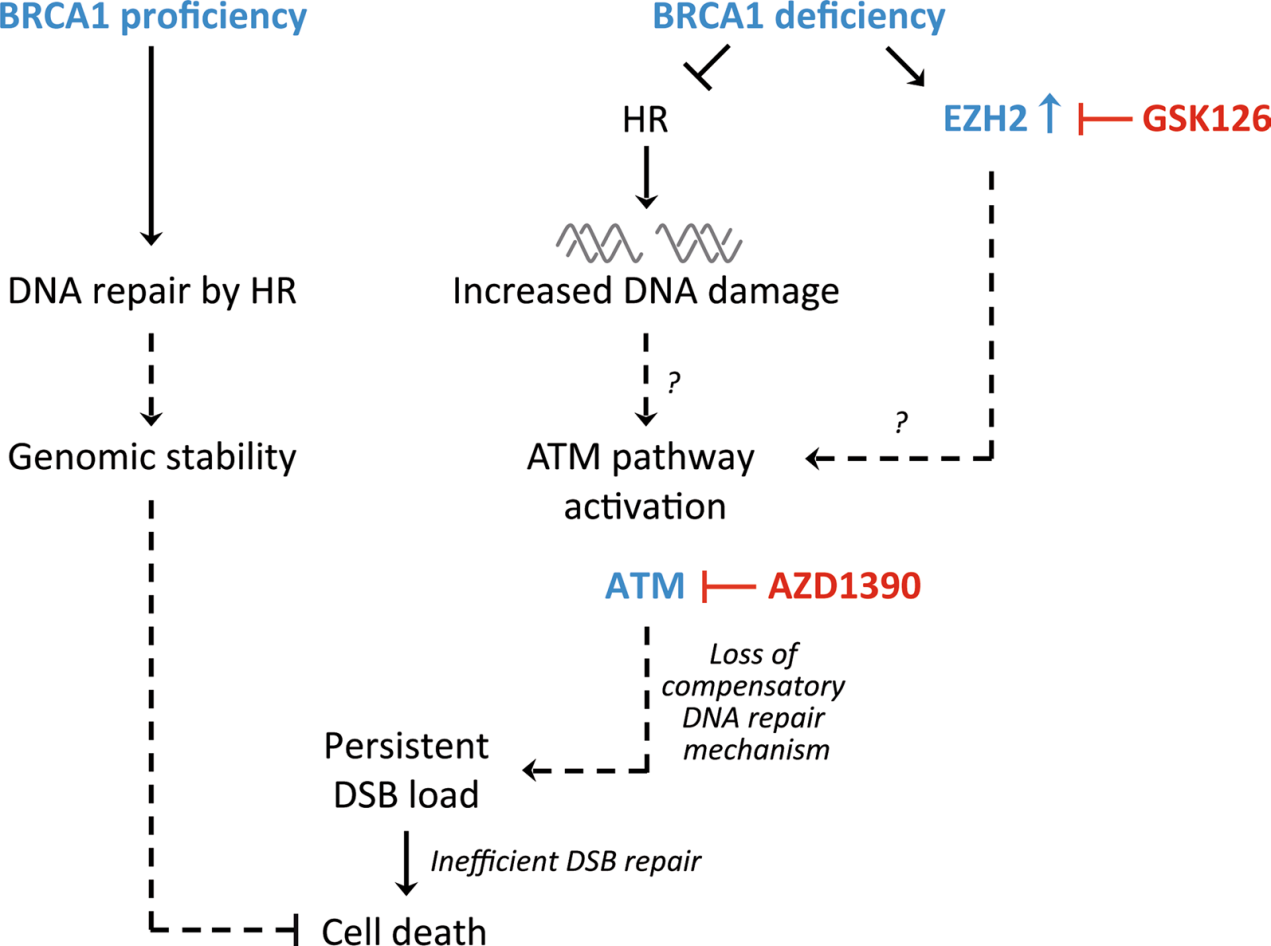
Proposed model for the synthetic lethal mechanism by combined EZH2/ATM inhibition for the treatment of BRCA1-deficient breast cancer. In BRCA1-deficient breast cancer cells, overexpression of EZH2 confers increased survival signaling. BRCA1-deficiency leads to compromised HR and accumulation of DNA DSBs. Simultaneous AZD1390-mediated ATM inhibition abolishes cell cycle checkpoint and compensatory DNA repair signaling leading to inefficient DSB repair, increased yH2AX foci and apoptosis-mediated cell death. Arrows and stop bars define activation and inhibition, respectively

Our data suggest that EZH2 inhibition can be combined with ATM inhibition to provoke toxic DSBs leading to apoptosis-mediated cell death in BRCA1-deficient breast cancer cells (Fig. 7). Xu et al. recently demonstrated that combined inhibition of EZH2 and ATM induced increased apoptosis in multiple myeloma cells [47]. The underlying mechanism of increased cytotoxicity by combined EZH2/ATM inhibition in BRCA1-deficient breast cancer cells is not clear. EZH2, as part of the PRC2 complex, can affect the cellular response to DNA damage in multiple ways. EZH2 is required for DNA damage-induced transcriptional silencing by PARP1-mediated EZH2 recruitment favoring DNA repair [48, 49], and EZH2 depletion conferred increased replicative stress [50, 51]. BRCA1/p53 double-deficient tumor cells are prone to increased replication stress and DSBs [10], which could explain, at least partly, the role of EZH2 inhibition as sensitizer to ATM inhibition (this manuscript) and cisplatin [14]. Further, EZH2-induced H3K27me3 methylation promotes chromatin compaction favoring reduced sensitivity to DNA-damage [47, 51, 52]. EZH2 inhibition or knockdown conferred a DNA damage-sensitivity phenotype to ionizing radiation and cisplatin [14, 48]. However, EZH2 was also demonstrated to negatively regulate RAD51 leading to increased sensitivity to the DNA-damaging effects of etoposide and ionizing radiation [53].

Since BRCA1-deficient cells experience higher levels of DNA damage, we assume that those cells are sensitive to ATM inhibition. Chen et al. suggested that ATM is essential for maintaining the residual levels of HR necessary to repair DSB in *BRCA1*-mutant cells [54]. Further, ATM signaling is a requirement for DDR signaling in response to genotoxic stress associated with BRCA1 deficiency [55]. In contrast to the findings by Chen et al. [54], our data show only moderate activity with AZD1390 single agent treatment at high doses in BRCA1-deficient cells.

Since EZH2 confers epigenetic silencing, it is tempting to speculate that inhibition of EZH2 methyltransferase activity would increase ATM expression. Naskou et al. found a correlation between low EZH2 and high ATM expression in ovarian cancer cells responsive to chemotherapy [56].They proposed that low EZH2 expression favors ATM overactivity to confer G2-M block, checkpoint arrest and potential resistance to chemotherapy [56]. Our proposed mechanism, although it remains speculative, could implicate additional replicative stress and an altered chromatin state induced by EZH2 inhibition in combination with the failure to resolve DSBs by ATM inhibition that leads to lethal levels of DNA damage in BRCA1/p53 double-deficient tumor cells. Considering the complex functions of EZH2, further aspects such as DNA repair pathway choice and expression of cell cycle regulators should also be taken into account.

Epigenetic therapies aim at reprogramming the aberrant epigenetic state rather than inducing cytotoxicity. Thus, a major advantage of this approach is that the effective dose level is below the maximal tolerated dose. Therefore, cytotoxic doses of EZH2 inhibitors can be avoided without losing the inhibitory effect on H3K27me3 levels. A limitation of our study is that the dose of AZD1390 used to induce synergistic cytotoxicity with GSK126 in vitro was higher than reported earlier [30] and normal tissue toxicity should be evaluated carefully in the context of clinical trials. In vivo, AZD1390 dose was well tolerated without significant toxicity to the animals.

Using *BRCA1* mutation status as predictive biomarker for targeted EZH2 treatment could circumvent a current major obstacle of rational clinical implementation of EZH2 inhibition since *EZH2* mutation status alone has been shown to be insufficient to stratify patients for therapy [57, 58]. Moreover, TNBC is an immunogenic tumor, however, BRCA1 mutation status has not yet been proven as predictive marker for immune checkpoint inhibition [59, 60]. The ability of combined EZH2/ATM inhibition to propagate DNA damage in BRCA1-deficient breast cancer could enhance the response to immuno-oncological treatment. Further investigation is necessary to elaborate the effect of combined EZH2/ATM inhibition on DNA damage-derived cytosolic DNA fragments that could trigger an anti-tumor immune response.

## Conclusion

We provide a rationalized approach of a synergistic therapy with EZH2 and ATM inhibition in BRCA1-deficient breast cancer that could guide further preclinical and clinical investigations. The identification of novel targeted treatment approaches is of high importance for patients with *BRCA1*-mutant breast cancer to overcome failure of a chemotherapy or resistance to PARP inhibition.

## Material and methods

### Integration with TCGA breast cancer patient data

Breast invasive carcinoma patient data from The Cancer Genome Atlas Breast Cancer (TCGA BRCA) were retrieved using the UCSC Cancer Browser (https://genome-cancer.ucsc.edu/). For graphical view of genomic data, whole-exome sequencing (*n* = 526) data were analyzed using the Xena browser at the cBioPortal (http://www.cbioportal.org). A detailed description of data generation and instructions can be viewed on https://xenabrowser.net [61].

### RNA sequencing

Gene expression was analyzed in KB1P and KP mouse mammary tumors. Illumina TruSeq mRNA libraries were generated and sequenced with 50–65 base single reads on a HiSeq 2500 using v4 chemistry (Illumina Inc., San Diego). The resulting reads were trimmed using Cutadapt (version 1.15) to remove any remaining adapter sequences and to filter reads shorter than 20 bp after trimming to ensure good mappability. The trimmed reads were aligned to the GRCm38 reference genome using STAR (version 2.6.1a [62]). Gene expression counts were generated by feautureCounts (version 1.5.0-p1 [63]) using genome definitions from Ensembl GRCm38 version 76. Normalized expression values were obtained by correcting for differences in sequencing depth between samples using DESeq median-of-ratios approach [64] and the log-transforming normalized counts. To statistically test the differences between the groups, ANOVA and pairwise *t*-test with multiple testing correction was used. The RNA sequencing data reported in this study are available in the NCBI GEO database (GSE182448).

### Cell lines and culturing

Murine tumor cell lines were generated from individual tumors arising in female KB1P or KP mice as described previously [29]. Established cell lines were cultured at 37 °C with 5% carbon dioxide under low oxygen conditions (3%) in DMEM/F12 medium (Thermo Fisher, Cat#31331028) supplemented with 10% FCS, 1% Penicillin–Streptomycin (5000 U/mL, Thermo Fisher, Cat#12140122), 5 mg/mL insulin (Sigma, Cat#53003-018), 5 ng/mL epidermal growth factor (Thermo Fisher, Cat#I6634), and 5 ng/mL cholera toxin (Sigma-Aldrich Israel (Cat#C8052).

KB1P-G3 and KP-3.33 cells stably expressing shEzh2 were cultured in DMEM/F12 medium supplemented with 10% FCS, 1% Penicillin–Streptomycin, 5 µg/mL insulin, 5 ng/mL epidermal growth factor, and 5 ng/mL cholera toxin. shRNA expression was induced with 100 ng/ml doxycycline (Dox) (Sigma-Aldrich, Cat#D9891) in culture medium. Medium of uninduced cells was supplemented with the respective volume of PBS.

Human SUM149 cells were cultured in DMEM/F12 medium supplemented with 10% FCS, 1% Penicillin–Streptomycin, 5 µg/mL insulin, and 1 µg/mL hydrocortisone (Sigma, Cat#0315). Human CAL120 cells were cultured in RPMI1640 (Thermo Fisher, Cat#12633012) supplemented with 10% FCS, 1% Penicillin–Streptomycin.

All cell lines were tested negative for Mycoplasma contamination upon thawing using a PCR Mycoplasma Test Kit (AppliChem, Cat#A3744).

### Pharmacological compounds

The following compounds were purchased from Selleckchem: AZD1390 (ATM inhibitor, Cat#S8680), AZD7762 (CHK1/2 inhibitor, Cat#S1532), BI2536 (PLK1 inhibitor, Cat#S1109), BKM120 (PI3K inhibitor, Cat#S2247), crizotinib (ROS1 inhibitor, Cat#S1068), dinaciclib (CDK inhibitor, Cat#S2768), gefitinib (EGFR inhibitor, Cat#S1025), GSK126 (EZH2 inhibitor, Cat#S7061), JQ1 (BET inhibitor, Cat#S7110), KU60019 (ATM inhibitor, Cat#S1570), KU60648 (DNA-PK inhibitor, Cat#S8045), LDC67 (CDK9 inhibitor, Cat#S7461), MK1775 (Wee1 inhibitor, Cat#S1525), olaparib (PARP inhibitor, Cat#S1060), palbociclib (CDK4/6 inhibitor, Cat#S1579), panobinostat (HDAC inhibitor, Cat#S1030), PF477736 (CHK1 inhibitor, Cat#S2904), purvalanol A (CDK1/2 inhibitor, Cat#S7793), RO3306 (CDK1 inhibitor, Cat#S7747), selisitat (SIRT1 inhibitor, Cat#S1541), selumetinib (MEK inhibitor, Cat#S1008), senexin A (CDK8/19 inhibitor, Cat#S8520), TH287 (MTH1/NUDT1 inhibitor, Cat#S7631), THZ1 (CDK7 inhibitor, Cat#S7549), VE822 (ATR inhibitor, Cat#S7102), venetoclax (BCL2 inhibitor, Cat#S8048). NSC663284 (Cdc25 inhibitor, Cat#383907-43-5) was purchased from Cayman Chemical, PF3644022 (MK2 inhibitor, Cat#B5549) from ApexBio, ZLD1039 (EZH2 inhibitor, Cat#AOB9716) from AOBIOUS. All compounds were dissolved in DMSO (Carl Roth, Cat#A994.2) at a concentration of 10 mM. Equal amounts of DMSO added to the cell culture medium served as vehicle control.

### Cell viability measurement

Optimal seeding density per cell line was derived from growth curves performed prior to screening experiments. Cells from subconfluent cell culture dishes were filtered through a cell strainer and viable cells were counted with Countess™ II FL Automated Cell Counter (Invitrogen) to allow optimal exponential growth (log phase) during the whole experiment. Cells were plated into 384-well plates (KB1P and KP cells: 500 cells/well, SUM149 and CAL120 cells: 1000 cells/well) in 30 µl complete culture medium. The compounds were added after 24 h by using the TECAN D300e digital dispenser (HP). After 72 h of treatment, cell viability was assessed by measuring ATP content in each well using CellTiter-Glo Reagent (Promega, Cat#G7573) 1:1. Luminescence intensity was measured using a plate reader (Tecan Infinite M1000 Pro) and normalized to intensities of control wells.

### Compound synergy screen

Before screening for drug synergy, single agent effects of all compounds were profiled on the KB1P and KP mouse mammary tumor cell lines for 10 different concentrations in two-fold serial dilutions, ranging from 20 nM to 20 µM and assessed by cell viability measurement using CellTiter-Glo. The concentration-effect relationship (IC_50_ values) was determined by logistic interpolation using GraphPad Prism. See Additional file 8: Table S1 for IC_50_ values.

Next, in a pre-screen for drug synergy, GSK126 was tested against each compound in two-fold serial dilutions to determine the optimal drug concentrations for synergy analysis using a 6 × 6 matrix for drug combinations (5 concentrations for each compound and DMSO control). For the main synergistic combination screen, 5 representative concentrations titrated around the previous determined IC_50_ values were used for each compound. The compounds were then profiled in combination with GSK126 using the 6 × 6 matrix layout, and cells were treated as described above See Additional file 8: Table S1 for compound concentrations. Cell viability was detected after 72 h using CellTiter-Glo Reagent. See Additional file 9: Table S2 for normalized cell viability data used for calculation of synergy scores.

### Analysis of synergy

Analysis of synergy scores was determined using the Bliss independence model calculating the difference between observed and expected compound effects [65] as described before [66]. Briefly, single agent effects of compounds A and B (*α*^A^, *α*^B^) at concentrations Cx^A^ and Cy^B^ were used to calculate the expected effect for additive compound interactions (*α*_exp_ = *α*^A^ + *α*^B^ − *α*^A^ * *α*^B^), and subsequently compared to the effect observed under combination of both compounds (*α*_obs_). The delta score (Δ*α* = *α*_obs_ − *α*_exp_) calculated as the difference between observed and expected effects over the full dose–response matrix characterizes the synergistic effect. A score of > 15 was used as threshold indicating a synergistic compound interaction [66]. Synergistic effects were visualized using the web-application tool SynergyFinder version 2.0 [32].

### Clonogenic survival assay and crystal violet staining

Cells were seeded at a density of 25,000 cells (KB1P and KP cells) or 50,000 cells (SUM149 and CAL120) per well into 6-well cell culture plates. After overnight incubation, cells were continuously treated with GSK126 (7.5 µM) or with AZD1390 (2 µM) alone or in combination for 7 days. DMSO was used as vehicle control.

KB1P-G3 and KP-3.33 cells stably expressing shEzh2 or shRandom were seeded into 10-cm cell culture dishes in the appropriate cell culture media 24 h before treatment. To induce *Ezh2* knockdown, cells were continuously treated with Dox (100 ng/µl) for 7 days. For the colony formation assay, cells treated with Dox for 7 days were replated at 10,000 cells per well into 12-well cell culture plates and cultivated in Dox for the duration of the entire experiment. Twenty-four hours after replating, cells were exposed to AZD1390 (2 µM) and incubated for 7 days in Dox-supplemented culture medium. Uninduced (PBS) and DMSO-treated cells were used as negative control.

After the treatment period, colonies were fixed with methanol on ice, stained with 0.5% crystal violet (Sigma-Aldrich, Cat#HT90132) and imaged using Carl Zeiss Stemi 2000-C Stereo microscope equipped with a CCD camera (Zeiss) at 0.65× magnification. Colonies were counted on whole surface area using the Clono-counter software and values were normalized to control wells [67].

### Real-time cell proliferation assay

Cells were plated into 384-well plates (500 cells/well) in 30 µl complete culture media. After overnight incubation, cells were treated with GSK126 (7.5 µM), AZD1390 (2 µM) or the combination of both. DMSO served as vehicle control. Cells were allowed to grow for 120 h. Phase-contrast images were automatically acquired by IncuCyte FLR (Essen Bioscience) from the incubator at 4-h intervals. Proliferation was monitored by analyzing the occupied area (% confluence) of cell images over time by IncuCyte software (Essen Bioscience).

### Cell lysis and immunoblotting

Whole-cell lysates were prepared in RIPA lysis buffer (50 mM Tris–HCl pH 7.5, 100 mM NaCl, 0.1% SDS, 0.5% Sodium deoxycholate) supplemented with 1 mM PMSF, 1% SDS, and phosphatase inhibitors (1x PhosphoSTOP Phosphatase Inhibitor Cocktail, Roche Diagnostics, Cat#4906845001). Samples were lysed on ice for 20 min, sheared by passing cells through a G25 needle (B. Braun, Hypodermic Needle-Pro®, Cat#4658304), cleared by centrifugation (14,000 × g, 15 min), and quantified using Pierce™ Bradford protein assay kit (Thermo Scientific, Cat#23200). Lysates were boiled 5 min at 95 °C with 6x reducing Laemmli buffer (0.12 M Tris pH 6.8, 47% glycerol, 12% SDS, 0.6 M DTT, 0.06% bromophenol blue). Samples were separated on a polyacrylamide gel and transferred to PVDF membranes using the mini wet/tank blotting system (Bio-Rad). After blocking with 5% BSA in Tween-20/PBS, membranes were probed with primary antibodies prepared in blocking solution overnight at 4 °C on a roller, followed by incubation with horseradish peroxidase-conjugated secondary antibody in blocking solution for 1 h at room temperature and ECL detection (Thermo Fisher, Cat#34096) by the ChemiDoc XRS + system (Bio-Rad). Primary and secondary antibodies used for immunoblotting are listed in Additional file 8: Table S1. Quantitative analysis of protein expression relative to GAPDH was done using Image Lab software (Bio-Rad).

### Analysis of apoptosis by flow cytometry

Cell lines (150,000 cells/well) were seeded into 6-well plates at 40–60% confluency. After overnight incubation, medium was replaced with growth medium containing single inhibitors GSK126 (7.5 µM) or AZD1390 (2 µM) or in combination for 48 h. Culture medium was collected, cells were trypsinized, washed with ice-cold PBS, and incubated with Annexin-V (BD Bioscience, Cat#556420) and propidium iodide (PI, Carl Roth, Cat#CN74, 5 µg/ml) in antibody binding buffer (2.5 mM CaCl_2_, 10 mM HEPES (pH 7.4), 140 mM NaCl, 20% accutase solution (Sigma-Aldrich, Cat#A6964), 70% PBS). Annexin-V/propidium iodide staining was detected by flow cytometry (Beckman Coulter, Gallios Flow Cytometer). The fraction of apoptotic cells was quantified as the Annexin-V, PI, and double positive stained populations using the Kaluza analysis software (Beckman Coulter).

### Immunofluorescence staining and high-throughput microscopy

Cells (5000 cells/well) were seeded into 96-well imaging plates (Greiner Bio-One, µclear, Cat#655090). After overnight incubation, cells were treated with single or combined agents of GSK126 (7.5 µM) and AZD1390 (2 µM). Treatment with 1 µM cisplatin was used as positive control. For pre-extraction of nonchromatin-bound proteins, after 48 h of treatment cells were incubated with sucrose buffer (25 mM HEPES pH 7.5, 50 mM NaCl, 1 mM EDTA, 3 mM MgCl_2_, 300 mM sucrose, 0.5% TritonX-100) for 2 min, followed by fixation in 4% paraformaldehyde for 10 min at RT. After 1 h of blocking (5% BSA, 2% normal goat serum, 0.1% TritonX-100, 0.05% Tween-20) at RT, cells were incubated with primary and secondary antibodies as listed in Additional file 8: Table S1. After three final washes, cells were covered with PBS and stored at 4 °C until scanning. Quantitative high-throughput microscopy was performed similarly as described before [66]. We used a Thermo Fisher Cellomics CellInsight CX7 LZR High Content Analysis (HCA) Platform with laser light source to scan stained cell models in 96-well imaging plates. 2 × 2 binned images (1104 × 1104 pixels) were acquired with a 20× (0.4 NA) Achroplan objective using the laser-based autofocus and analyzed using the Spotdetector V4.1 Bioapplication of the Cellomics software package (Version 6.6.2, Built 8533). Cell nuclei were identified by Hoechst 34580 staining in background corrected images (3D surface fitting) according to the object identification parameters size: 100–1500 μm^2^, ratio of perimeter squared to 4π area: 1–5, length-to-width ratio: 1–5, average intensity: 500–8000, total intensity: 2x105–5x107. Foci were identified within the nuclear region using the Box method with a value of 3. Object selection parameters for foci were 1–30 μm^2^, ratio of perimeter squared to 4π area: 1–5, length-to-width ratio: 1–5, average intensity: 500–16,000, total intensity: 3x102–1x106.

### Orthotopic tumor transplantation and in vivo drug intervention study

This study is compliant with all relevant ethical regulations regarding animal research. All animal experiments were approved by the Animal Ethics Committee of The Netherlands Cancer Institute (Amsterdam, the Netherlands) and performed in accordance with the Dutch Act on Animal Experimentation. Generation of conditional K14*cre*; *Brca1*^F/F^; *Trp53*^F/F^ (KB1P) and K14*cre*; *Trp53*^F/F^ (KP) breast cancer mouse models were described previously [68, 69]. Orthotopic transplantations, tumor monitoring, and tissue sampling were performed as described before [70]. Briefly, donor KB1P tumor fragments were transplanted into the fourth mammary fat pad of 8-weeks old FVB females and treatments began upon reaching tumor outgrowth of approximately 100 mm^3^ (100%). Maximal tolerable dose (MTD) for the combined therapy with GSK126 and AZD1390 was determined prior to the intervention study using FVB females. GSK126 (Syncom) was reconstituted in 20% Captisol (CyDex Pharmaceuticals) and brought to a pH of 4.5 with 10 M potassium hydroxide, to create a working stock of 15 mg/mL. AZD1390 (Selleckchem) was dissolved at 40 mg/ml in ethanol and then dropwise added to 0.5% (w/v) HPMC, 0.1% (w/v) Tween-80 to achieve a final concentration of 2 mg/ml. Tumor-bearing mice were blindly randomized into four treatment groups and treated with vehicle (daily intraperitoneal injection), GSK126 (150 mg/kg, daily intraperitoneal injection), AZD1390 (bi-daily 20 mg/kg, by oral gavage for 5 days on, 2 days off) or a combination of GSK126 and AZD1390. Animals were treated for 28 consecutive days and mammary tumor volume (mm) was quantified by caliper measurements using the following formula: 0.5 × length × width^2^. For a better comparability, the tumor volume at day x was normalized to the initial tumor volume at treatment start (day 0) and defined as relative tumor volume (RTV). The endpoint of this study was reached when tumor size was 10 times the RTV, defined as progression-free survival (PFS). Animals were euthanized by CO_2_ when tumors extended a volume of 1500 mm^3^ or when severe side effects were observed. All procedures were carried out by animal technicians in a blinded fashion.

### In vivo target inhibition by GSK126 and AZD1390

A small cohort of mice (*n* = 12) was sacrificed after 7 days of single agent or combined treatment with GSK126 and AZD1390, and KB1P tumors were harvested for immunohistochemistry analysis (IHC). IHC staining of EZH2, H3K27me3, and phosphorylated ATM was performed using formalin-fixed paraffin-embedded tumor tissue. The following monoclonal antibodies were used for immunohistochemistry: EZH2 (Rb, Cell signaling, Cat#5246, 1:200), H3K27me3 (Rb, Abcam, Cat#ab6002, 1:100) and phosphorylated ATM (phospho S1981, Rb, Abcam, Cat#ab81292, 1:400) overnight at 4 °C. These antibodies were extensively tested for target specificity [14]. All slides were digitally processed using the Aperio ScanScope (Aperio, Vista, CA, USA) and captured using ImageScope software version 12.0.0 (Aperio).

### Statistical analysis

Statistical analysis was performed using GraphPad Prism Version 8 and 9. Mann–Whitney U test, one-way ANOVA with Tukey multiple comparison testing or Kaplan–Meier survival testing with log-rank comparison were used as indicated in the figure legends. *p* values below 0.05 were considered statistically significant. Significance levels are indicated as: *—*p* ≤ 0.05; **—*p* ≤ 0.01; ***p ≤ 0.001; nonsignificant levels are not labeled. Number of experimental replicates are indicated in the figure legends.

## Supplementary Information

**Additional file 1: Fig. S1.** Single agent dose-response curves and IC_50_ determination. (**A**) Single agent cytotoxicity of compounds was evaluated in two BRCA1-deficient (KB1P-G3 and KB1P-B11) and two BRCA1-proficient (KP-3.33 and KP-6.3) mouse mammary tumor cell lines. Cells were permanently exposed to 10 different compound concentrations ranging from 20 nM to 20 µM for 72 hours. Cell viability was determined by measuring ATP content using CellTiter-Glo assay. The IC_50_ values were determined by logistic interpolation using GraphPad Prism software. Dotted lines represent IC_50_ concentration in the graph. See Additional file 8: Table S1 for IC_50_ values for the compounds and four different cell lines.

**Additional file 2: Fig. S2.** Synergy heatmaps for compound combinations. Heatmaps visualized by the web-application tool SynergyFinder showing synergy scores of 25 compounds each in combination with GSK126 after 72 hours treatment. Displayed colors reflect the growth inhibition in percent with red indicating stronger inhibition and green indicating lower inhibition. Combinations are arranged by the difference in synergy score between BRCA1-deficient and BRCA1-proficient cell lines from high to low. See Additional file 8: Table S1 for individual synergy scores and Additional file 9: Table S2 for normalized cell viability data used for calculation of synergy scores.

**Additional file 3: Fig. S3.** Concentration optimization for combined GSK126/AZD1390 treatment. Determination of optimized synergistic compound concentrations for the prioritized drug combination GSK126/AZD1390 resulting in maximal synergistic effects. Cells were permanently exposed to single agent or combined treatment as indicated. After 72 hours treatment, cell viability was measured using CellTiter-Glo assay. (**A**) Dose-response curves of increasing concentrations of GSK126 (1.25, 2.5, 5, 7.5, 10 µM) alone (orange) or in combination with a fixed concentration of AZD1390 (1, 2, 3, 4, 5 µM) (purple). (**B**) Dose-response curves of AZD1390 (1, 2, 3, 4, 5 µM) alone (orange) or in combination with a fixed concentration of GSK126 (1.25, 2.5, 5, 7.5, 10 µM) (purple). Black arrows indicate inhibitor concentrations with maximal synergistic effect after 72 hours treatment. Statistical significance was tested by one-way ANOVA with Tukey multiple comparison test.

**Additional file 4: Fig. S4.** Combined inhibition of EZH2/ATM induces genotoxic stress. Bar graphs show cells per field (upper panels) and number of analyzed cells per treatment condition (lower panels) after treatment with 7.5 µM GSK126 and 2 µM AZD1390 for 48 hours presented as mean ± SEM of at least four independent experiments with each three technical replicates.

**Additional file 5: Fig. S5.** Immunoblotting showing ATM signaling upon GSK126/AZD1390 treatment. The data show inhibition of phospho-ATM and downstream phospho-Kap1 after AZD1390 single and combination treatment with GSK126, which is the expected effect of AZD1390-mediated ATM inhibition. Cisplatin served as positive control showing increased levels of phospho-Kap1.

**Additional file 6: Fig. S6.** Drug dose optimization for in vivo treatment. Mammary tumor tissue fragments from KB1P mice were transplanted into the fourth mammary fat pad of FVB females and treatments were initiated following tumor outgrowth to approximately 100 mm^3^ (100%). Upon tumor detection (day 0), mice were treated for 28 consecutive days. For drug dose optimization, we used GSK126 75 mg/kg daily by intraperitoneal injection (dotted line), AZD1390 once-daily 20 mg/kg, by oral gavage for 5 days on, 2 days off (dashed line), combined treatment with GSK126/AZD1390 (black)or vehicle (gray). Here, were observed a mild effect on progression-free survival in the combination arm. The combination was well-tolerated. To improve the efficacy of the combination therapy, we doubled the doses for the final experiment to the highest tolerable dose: GSK126 (150 mg/kg, daily intraperitoneal injection), AZD1390 (twice-daily 20 mg/kg, by oral gavage for 5 days on, 2 days off).

**Additional file 7: Fig. S7.** Drug tolerability in vivo: Body weight measurements during drug treatment and censored animals. KB1P mammary tumor-bearing mice treated with vehicle (gray), GSK126 (150 mg/kg i.p.) single agent (dotted line), AZD1390 (2 x 20 mg/kg, oral gavage) single agent (dashed line), or combined treatment with GSK126/AZD1390 (black) for 28 consecutive days. Weight was measured daily before drug administration, and depicted as mean ± SEM. Censored animals: 3 mice were lost during the experiment: 1 mouse was found dead in cage in the combination arm after 4 days of treatment; 1 mouse was found dead in cage in the combination arm after 20 days of treatment; 1 mouse had to be sacrificed due to open tumor in the combination arm on day 16. Two mice were replaced during the experiment in the combination arm.

**Additional file 8: Table S1**. Table S1 complements the Material and Methods part listing IC_50_ values of single compounds (data presented in Additional file 2: Fig. S2), compound concentrations, calculated synergy scores and antibodies.

**Additional file 9: Table S2**. Table S2 contains normalized cell viability values of 6x6 combination experiments (original data of Fig. 1 and Additional file 2: Fig. S2).

## Data Availability

All data generated or analyzed during this study are included in the published article and its additional information.

## Abbreviations

ATM: Ataxia telangiectasia mutated
DSB: Double strand break
EZH2: Enhancer of zeste homolog 2
gBRCA: Germline *BRCA1/2* mutation-associated breast cancer
HR: Homologous recombination
NHEJ: Non-homologous end joining
TCGA BRCA: The Cancer Genome Atlas Breast Invasive Carcinoma
TNBC: Triple-negative breast cancer
