# Supplementary figures and images for: Combined inhibition of EZH2 and ATM is synthetic lethal in BRCA1-deficient breast cancer
Source: Breast Cancer Res. 2022 Jun 17;24:41. doi: 10.1186/s13058-022-01534-y (PMC9206299; doi:10.1186/s13058-022-01534-y)

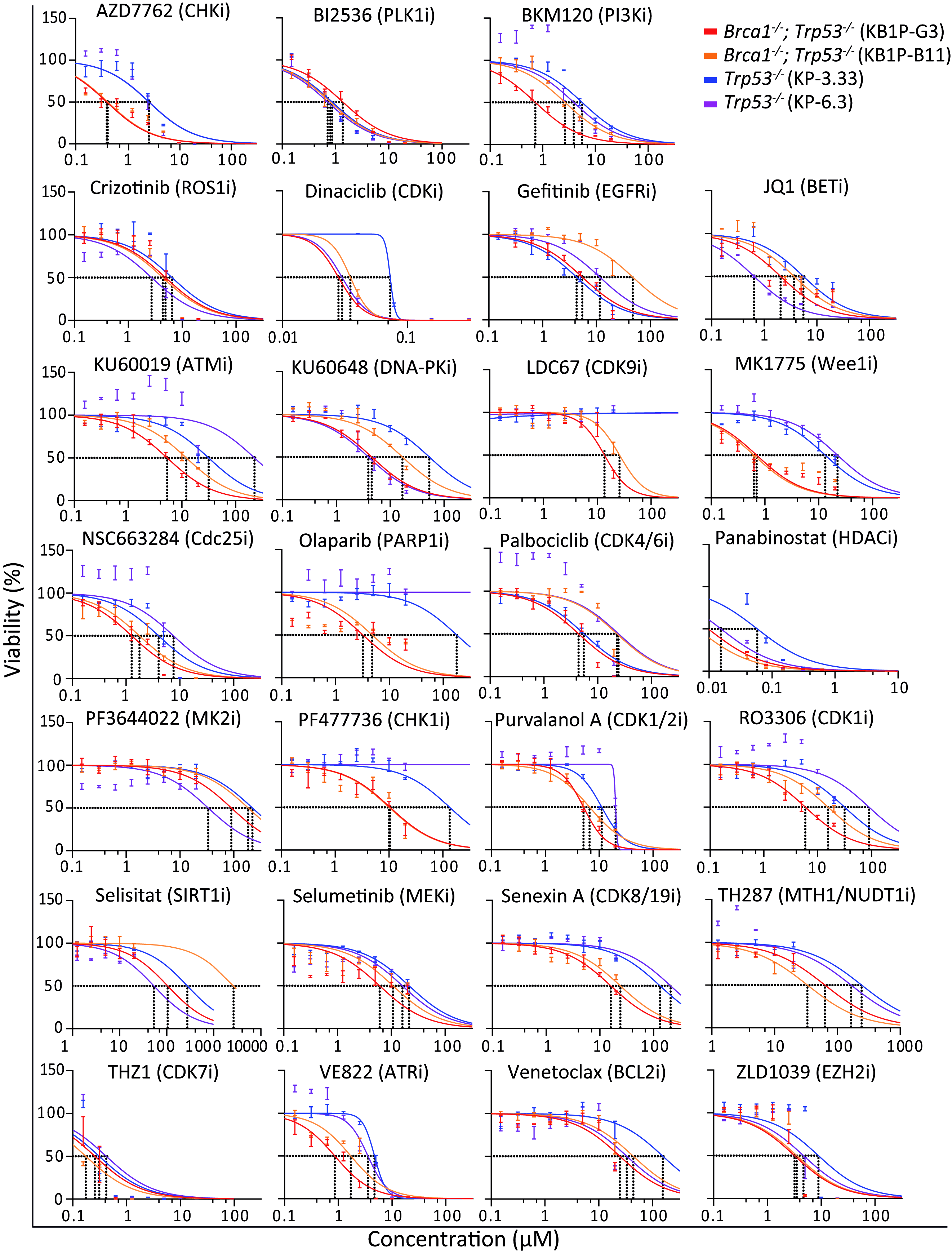

Supplement: Supplementary file 1 — Additional file 1: Fig. S1. Single agent dose-response curves and IC50 determination. (A) Single agent cytotoxicity of compounds was evaluated in two BRCA1-deficient (KB1P-G3 and KB1P-B11) and two BRCA1-proficient (KP-3.33 and KP-6.3) mouse mammary tumor cell lines. Cells were permanently exposed to 10 different compound concentrations ranging from 20 nM to 20 µM for 72 hours. Cell viability was determined by measuring ATP content using CellTiter-Glo assay. The IC50 values were determined by logistic interpolation using GraphPad Prism software. Dotted lines represent IC50 concentration in the graph. See Additional file 8: Table S1 for IC50 values for the compounds and four different cell lines. [file 13058_2022_1534_MOESM1_ESM.tif]

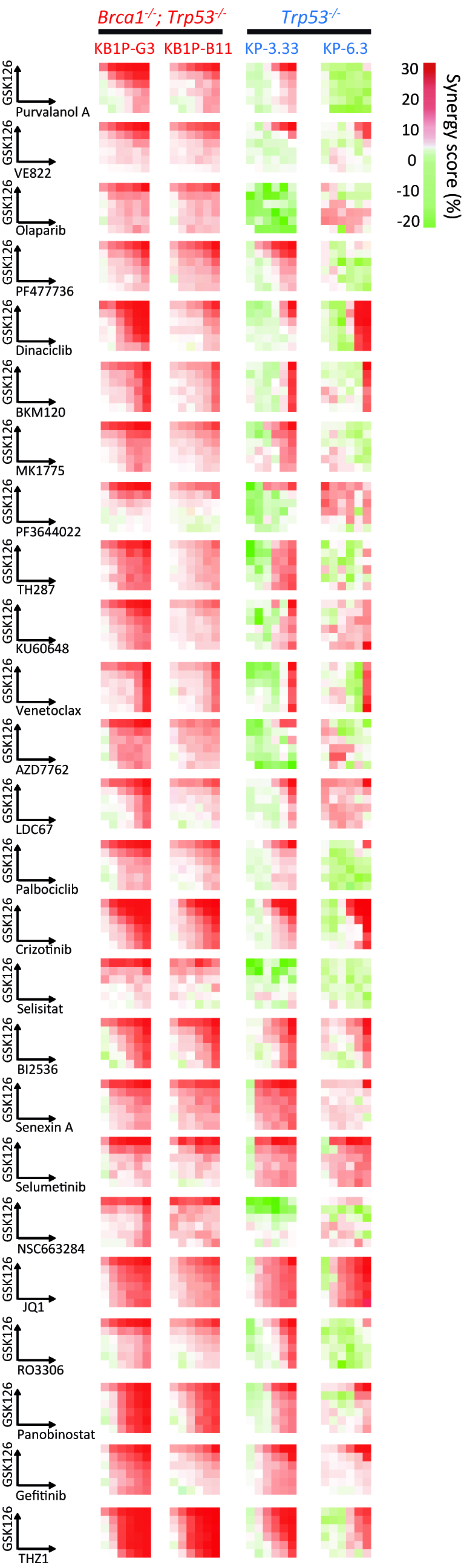

Supplement: Supplementary file 2 — Additional file 2: Fig. S2. Synergy heatmaps for compound combinations. Heatmaps visualized by the web-application tool SynergyFinder showing synergy scores of 25 compounds each in combination with GSK126 after 72 hours treatment. Displayed colors reflect the growth inhibition in percent with red indicating stronger inhibition and green indicating lower inhibition. Combinations are arranged by the difference in synergy score between BRCA1-deficient and BRCA1-proficient cell lines from high to low. See Additional file 8: Table S1 for individual synergy scores and Additional file 9: Table S2 for normalized cell viability data used for calculation of synergy scores. [file 13058_2022_1534_MOESM2_ESM.tif]

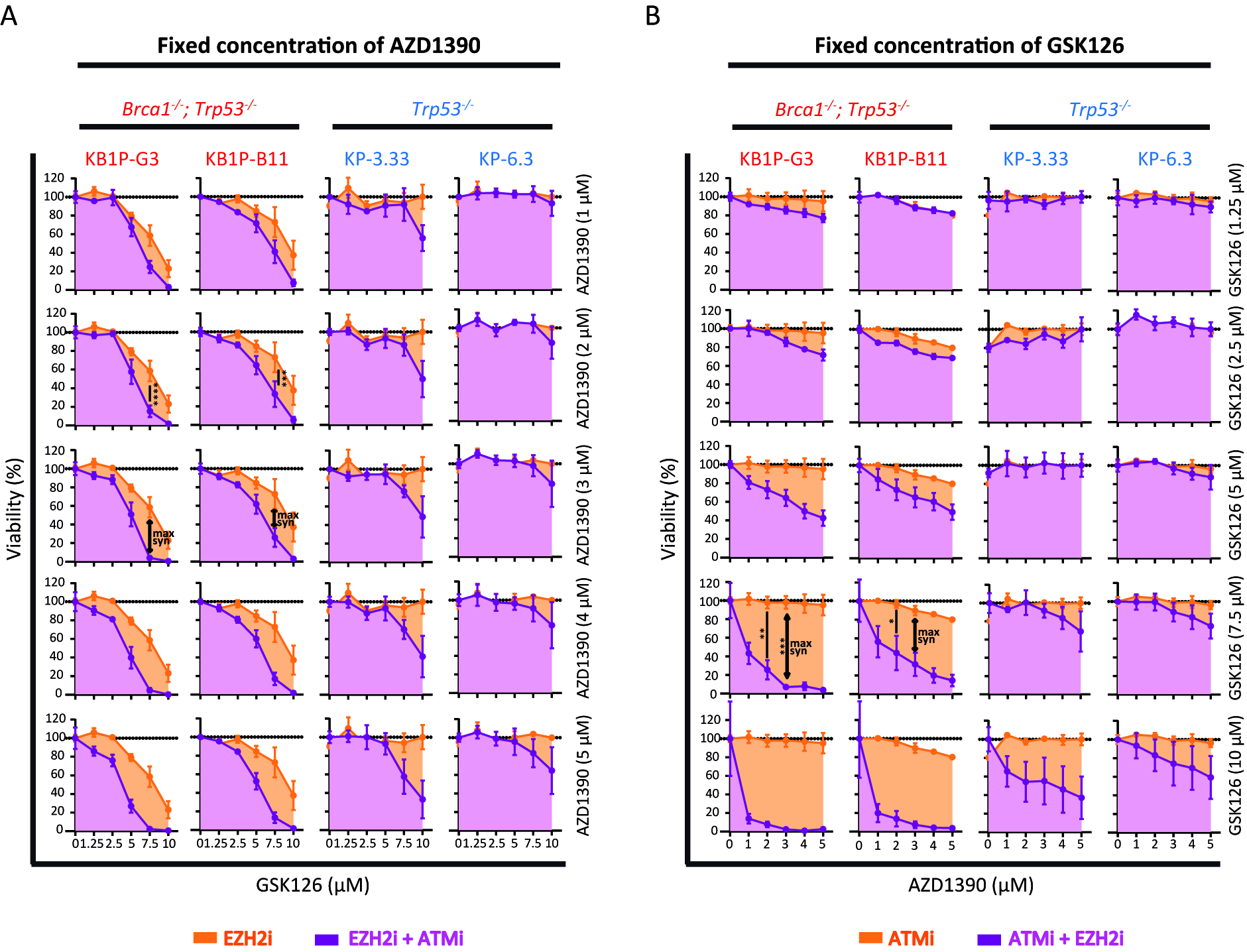

Supplement: Supplementary file 3 — Additional file 3: Fig. S3. Concentration optimization for combined GSK126/AZD1390 treatment. Determination of optimized synergistic compound concentrations for the prioritized drug combination GSK126/AZD1390 resulting in maximal synergistic effects. Cells were permanently exposed to single agent or combined treatment as indicated. After 72 hours treatment, cell viability was measured using CellTiter-Glo assay. (A) Dose-response curves of increasing concentrations of GSK126 (1.25, 2.5, 5, 7.5, 10 µM) alone (orange) or in combination with a fixed concentration of AZD1390 (1, 2, 3, 4, 5 µM) (purple). (B) Dose-response curves of AZD1390 (1, 2, 3, 4, 5 µM) alone (orange) or in combination with a fixed concentration of GSK126 (1.25, 2.5, 5, 7.5, 10 µM) (purple). Black arrows indicate inhibitor concentrations with maximal synergistic effect after 72 hours treatment. Statistical significance was tested by one-way ANOVA with Tukey multiple comparison test. [file 13058_2022_1534_MOESM3_ESM.tif]

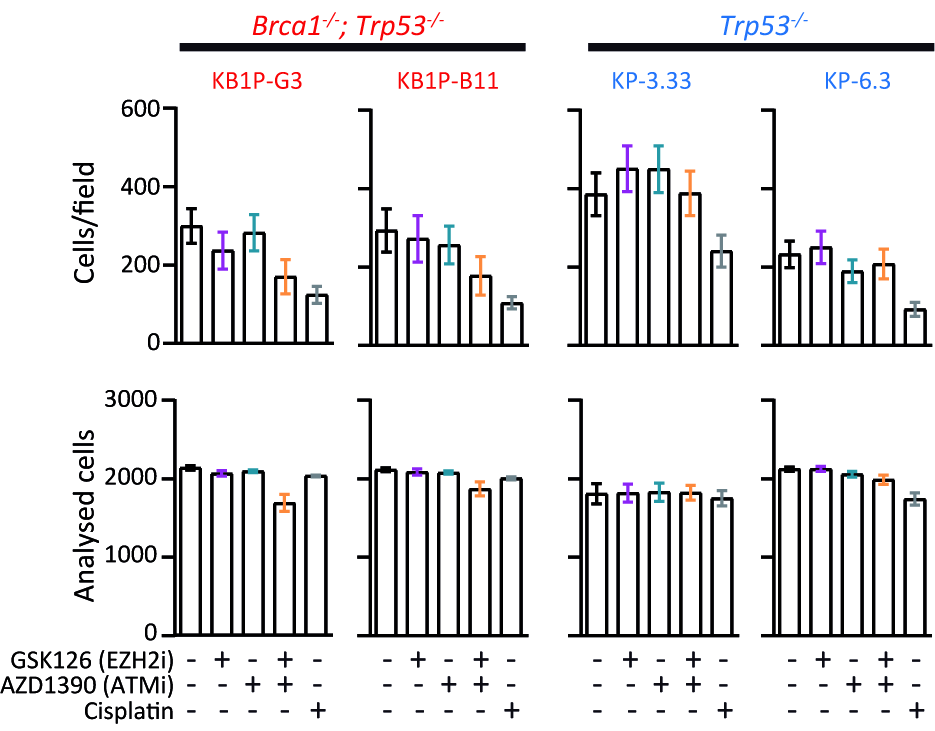

Supplement: Supplementary file 4 — Additional file 4: Fig. S4. Combined inhibition of EZH2/ATM induces genotoxic stress. Bar graphs show cells per field (upper panels) and number of analyzed cells per treatment condition (lower panels) after treatment with 7.5 µM GSK126 and 2 µM AZD1390 for 48 hours presented as mean ± SEM of at least four independent experiments with each three technical replicates. [file 13058_2022_1534_MOESM4_ESM.tif]

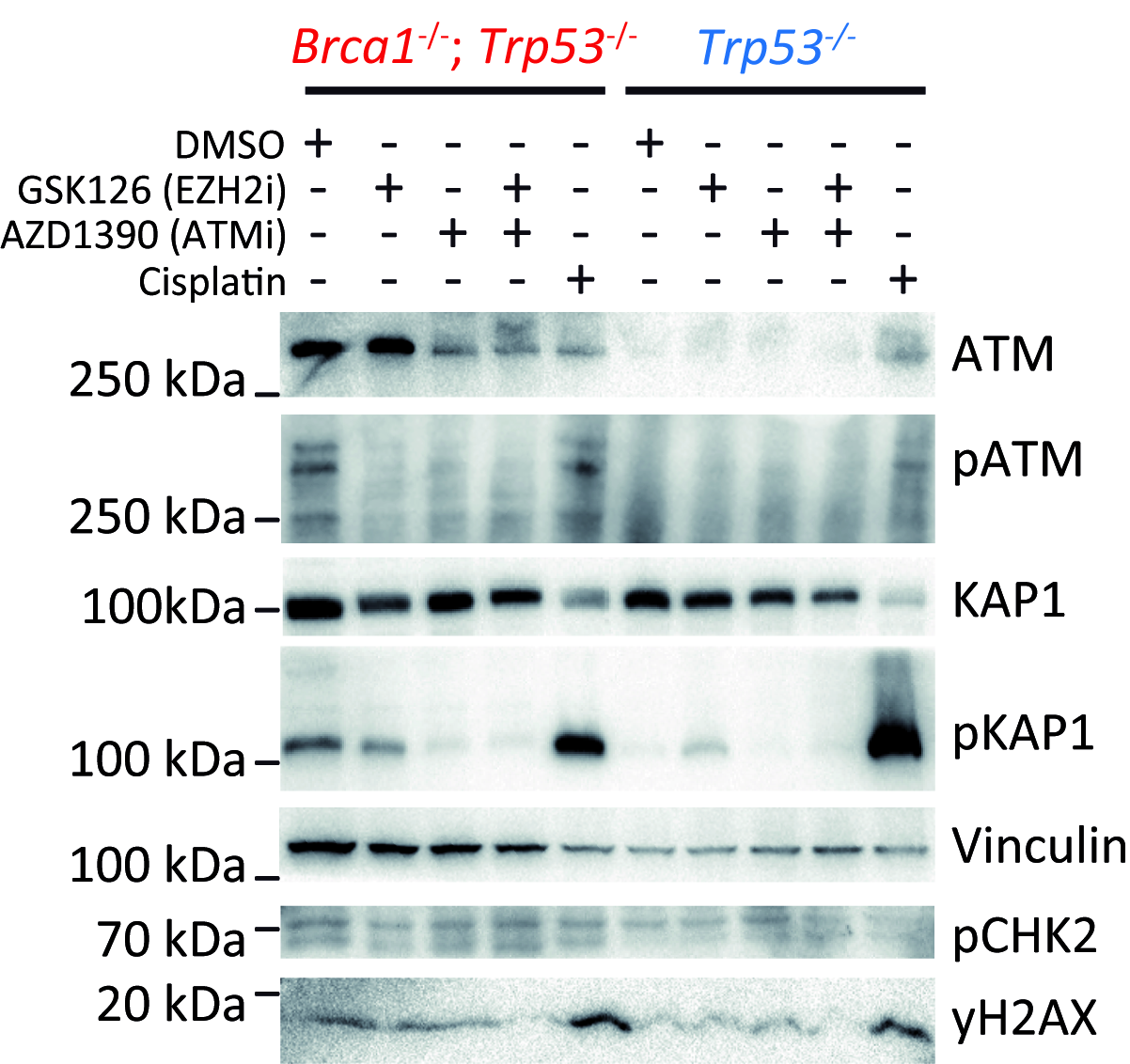

Supplement: Supplementary file 5 — Additional file 5: Fig. S5. Immunoblotting showing ATM signaling upon GSK126/AZD1390 treatment. The data show inhibition of phospho-ATM and downstream phospho-Kap1 after AZD1390 single and combination treatment with GSK126, which is the expected effect of AZD1390-mediated ATM inhibition. Cisplatin served as positive control showing increased levels of phospho-Kap1. [file 13058_2022_1534_MOESM5_ESM.tif]

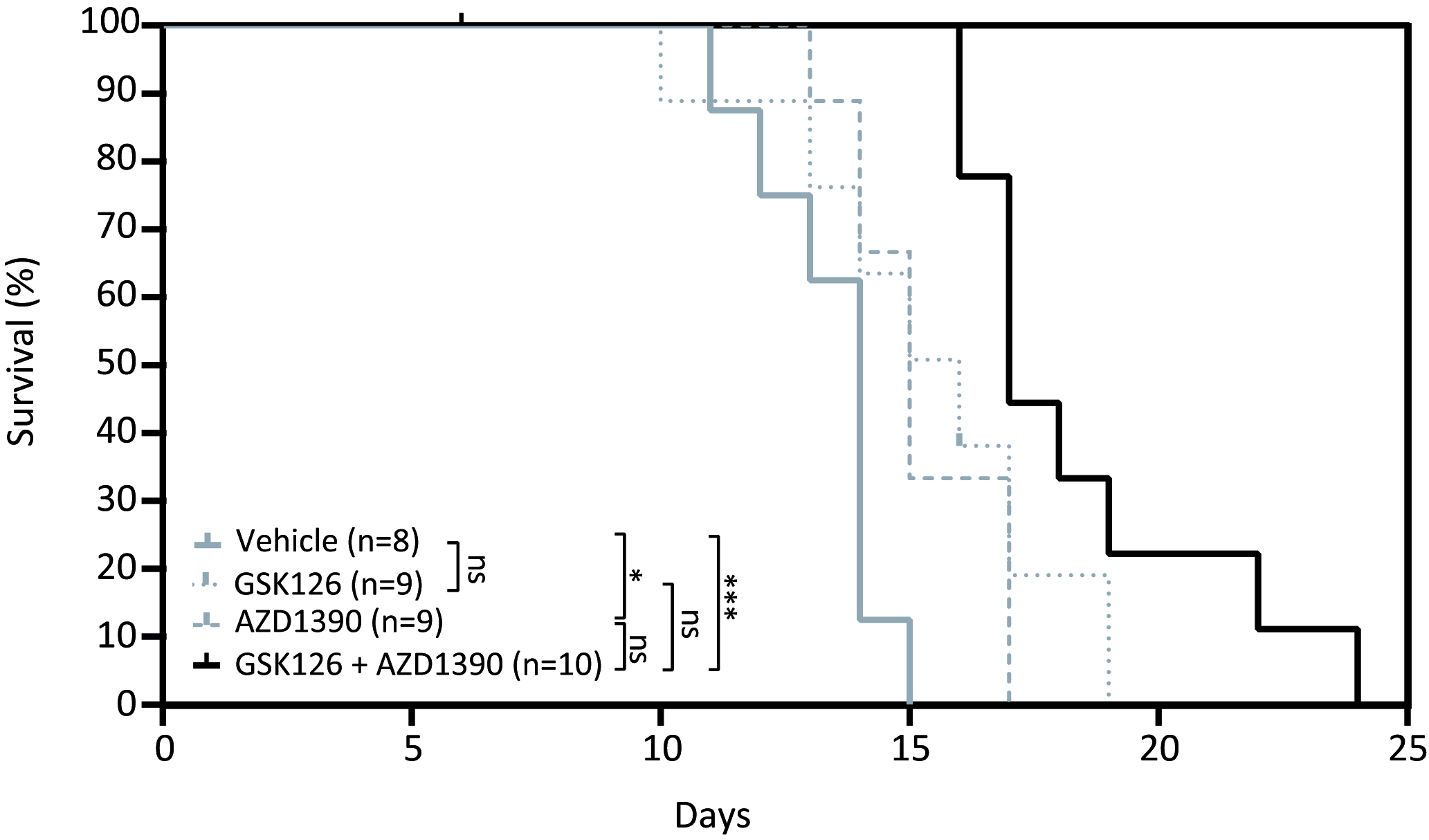

Supplement: Supplementary file 6 — Additional file 6: Fig. S6. Drug dose optimization for in vivo treatment. Mammary tumor tissue fragments from KB1P mice were transplanted into the fourth mammary fat pad of FVB females and treatments were initiated following tumor outgrowth to approximately 100 mm3 (100%). Upon tumor detection (day 0), mice were treated for 28 consecutive days. For drug dose optimization, we used GSK126 75 mg/kg daily by intraperitoneal injection (dotted line), AZD1390 once-daily 20 mg/kg, by oral gavage for 5 days on, 2 days off (dashed line), combined treatment with GSK126/AZD1390 (black)or vehicle (gray). Here, were observed a mild effect on progression-free survival in the combination arm. The combination was well-tolerated. To improve the efficacy of the combination therapy, we doubled the doses for the final experiment to the highest tolerable dose: GSK126 (150 mg/kg, daily intraperitoneal injection), AZD1390 (twice-daily 20 mg/kg, by oral gavage for 5 days on, 2 days off). [file 13058_2022_1534_MOESM6_ESM.tif]

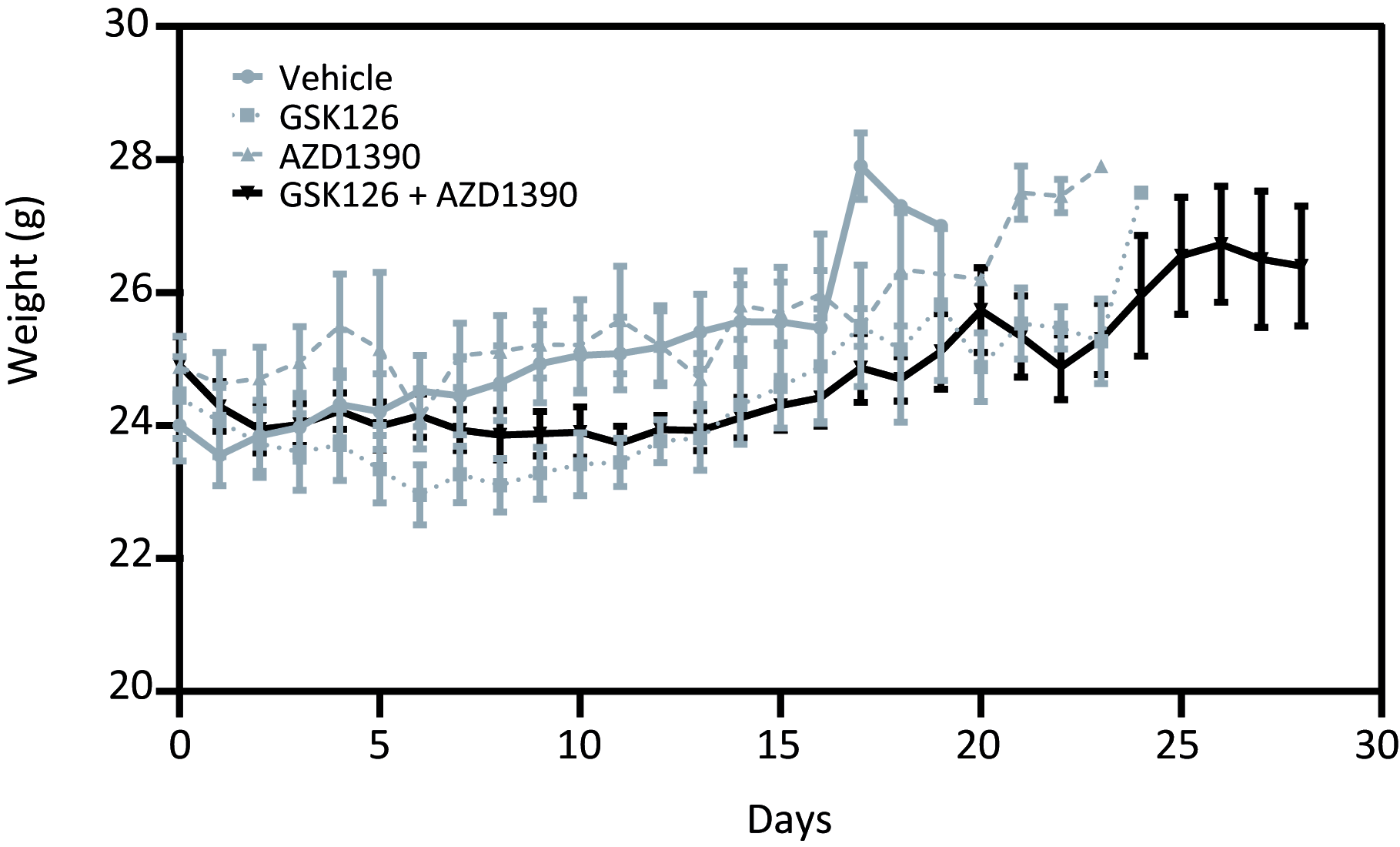

Supplement: Supplementary file 7 — Additional file 7: Fig. S7. Drug tolerability in vivo: Body weight measurements during drug treatment and censored animals. KB1P mammary tumor-bearing mice treated with vehicle (gray), GSK126 (150 mg/kg i.p.) single agent (dotted line), AZD1390 (2 x 20 mg/kg, oral gavage) single agent (dashed line), or combined treatment with GSK126/AZD1390 (black) for 28 consecutive days. Weight was measured daily before drug administration, and depicted as mean ± SEM. Censored animals: 3 mice were lost during the experiment: 1 mouse was found dead in cage in the combination arm after 4 days of treatment; 1 mouse was found dead in cage in the combination arm after 20 days of treatment; 1 mouse had to be sacrificed due to open tumor in the combination arm on day 16. Two mice were replaced during the experiment in the combination arm. [file 13058_2022_1534_MOESM7_ESM.tif]
